# Alleviation of heat stress-induced microbial dysbiosis in pigs through dietary supplementation with vitamins and trace elements

**DOI:** 10.1186/s42523-026-00575-4

**Published:** 2026-04-21

**Authors:** Peter Fauszt, Endre Szilagyi, Maja Mikolas, Emese Szilagyi-Tolnai, Peter David, Ildiko Noemi Kovacs-Forgacs, Brigitta Csernus, Ferenc Gal, Laszlo Stundl, Sandor Biro, Csaba Szabo, Judit Remenyik, Laszlo Babinszky, Melinda Paholcsek

**Affiliations:** 1 Faculty of Agricultural and Food Sciences and Environmental Management, University of Debrecen, Complex Systems and Microbiome-innovations Centre, Debrecen, Hungary; 2 Department of Evolutionary Zoology and Human Biology, Debrecen, Hungary; 3 Faculty of Agricultural and Food Sciences and Environmental Management, University of Debrecen, Institute of Food Technology, Debrecen, Hungary; 4 Faculty of Medicine, Department of Human Genetics, University of Debrecen, Debrecen, Hungary; 5 Faculty of Agricultural and Food Sciences and Environmental Management, Institute of Animal Science, Biotechnology and Nature Conservation, Department of Animal Nutrition and Physiology, University of Debrecen, Debrecen, Hungary

## Abstract

**Background:**

Chronic heat stress (HS) is known to impair animal health and productivity, in part by altering gut microbiota. This study investigated how HS affects the pig gut microbiome and whether dietary supplementation with antioxidants and trace elements (vitamins E, C, selenium, and zinc) at moderate (D1) or high (D2) doses can mitigate these effects.

**Results:**

During the adaptation phase, feed efficiency was similar across groups, but as the experiment progressed, the thermoneutral control improved while the heat-stressed control deteriorated. Supplemented diets (D1/D2) partially alleviated this efficiency loss. Microbiome analysis revealed that HS progressively reduced diversity, reaching the lowest Shannon index during exposure. High-dose supplementation markedly increased richness, exceeding control levels. Total microbial abundance declined under HS, with opportunistic pathogens enriched particularly during early exposure. Guild-level indices further indicated a shift under HS. Aerotolerance indices decreased (ATi: TC > D1 > D2 > HSC), reflecting hypoxia-prone conditions favoring obligate anaerobes and SCFA producers. Among supplemented groups, D1 most closely stabilized aerotolerance toward control levels, while D2 maintained an SCFA-dominant community and enhanced butyrate capacity. Genus-level correlations with qPCR-based host gene-expression markers were assessed across all groups. HSP70 was the dominant correlate, and the most extreme associations were confined to a few taxa, indicating marked group specificity.

**Conclusion:**

Chronic HS in pigs induced microbial dysbiosis characterized by reduced diversity, loss of beneficial SCFA producers, and expansion of opportunistic pathogens. Dietary supplementation counteracted these adverse changes in a dose-dependent manner. While moderate supplementation provided partial stabilization, high-dose supplementation more effectively restored microbial diversity and enriched beneficial taxa, making it the more effective strategy for mitigating HS-induced microbiome disruption.

**Supplementary Information:**

The online version contains supplementary material available at 10.1186/s42523-026-00575-4.

## Background

Heat stress is widely recognized as a major environmental stressor in livestock, impairing feed intake, growth performance, reproductive efficiency, and immune function while increasing oxidative stress and systemic inflammation. In addition, elevated ambient temperatures disrupt intestinal barrier integrity and alter gut microbiota composition, contributing to dysbiosis and increased susceptibility to disease [1].

Heat stress arises when ambient temperatures exceed the thermoneutral zone, impairing the animal’s ability to dissipate excess heat and leading to cascading effects on welfare, immune function, and performance [2, 3]. Heat stress is a condition that occurs when the body is unable to properly regulate its internal temperature, causing the body’s core temperature to rise and heat to build up [4]. It is the cumulative result of environmental factors (high temperature, humidity, radiant heat) and internal heat generated by physical activity. When the body’s cooling mechanisms, such as sweating and increased blood flow to the skin, are overwhelmed, it leads to a state where the body cannot shed excess heat, potentially leading to serious heat-related illnesses [4].

Pigs are particularly susceptible to thermal stress due to their inefficient thermoregulatory physiology. They possess few functional sweat glands and are typically unable to engage in self-wetting behaviors under conventional housing conditions, severely limiting evaporative heat loss [5]. As a result, pigs experience thermoregulatory failure at relatively moderate ambient temperatures compared to other livestock species.

In Hungary, the pig sector faces growing vulnerability as climate change accelerates. Summers are increasingly marked by extended heat waves and irregular rainfall, contributing to both physiological stress and infrastructural strain. Heat stress has emerged as a critical disruptor of animal health and productivity, particularly under the intensive housing conditions common in the Central and Eastern European (CEE) region [2].

These pressures exacerbate existing productivity challenges by reducing feed intake, impairing feed conversion ratio (FCR), and lowering reproductive performance. Studies report reductions in FCR among finishing pigs and 5–10% declines in sow fertility during peak heat periods [6].

Beyond systemic effects, heat stress induces pronounced disruptions in gut physiology and microbial ecology. One of the earliest consequences is the compromise of intestinal epithelial integrity, resulting in increased paracellular permeability or “leaky gut.” This facilitates the translocation of luminal pathogens, endotoxins, and microbial metabolites into systemic circulation, triggering inflammation and immune dysregulation.

Simultaneously, elevated temperatures drive compositional shifts in the gut microbiota, including reduced microbial diversity and depletion of beneficial taxa such as *Lactobacillus*, *Bifidobacterium*, and short-chain fatty acid (SCFA)-producing genera like *Faecalibacterium*. Dysbiosis impairs nutrient absorption, weakens mucosal immunity, and increases the risk of opportunistic infections [7, 8]. Notably, researchers reported increased prevalence and virulence of enteric pathogens, particularly *Escherichia coli* and *Salmonella* spp., in heat-stressed pigs, raising concerns for both animal health and public food safety through elevated environmental shedding and zoonotic risk [9, 10].

The gut microbiota plays a pivotal role in maintaining host resilience under environmental stress. Through the production of short-chain fatty acids, bile acid metabolites, and other bioactive signaling molecules, the microbiota modulates epithelial integrity, immune responses, and nutrient metabolism [11, 12]. Maintaining microbial diversity and functional capacity is thus essential for sustaining performance and health in thermally challenged pigs.

Given these interdependencies, microbiome-informed nutritional strategies have gained prominence. Feed additives such as probiotics, postbiotics, paraprobiotics, and targeted micronutrients (e.g., vitamins and trace elements) are increasingly adopted to enhance host resilience, reduce pathogen burden, and improve performance under stress without substantially increasing production costs [13–15].

While the effects of acute heat stress on pig physiology are well documented, fewer studies have addressed how chronic thermal exposure alters gut microbial composition and function under production-relevant conditions. Moreover, the capacity of targeted nutritional interventions to mitigate these effects through microbiota modulation remains insufficiently characterized, particularly in climate-vulnerable regions such as Central and Eastern Europe [16].

Based on the central role of the gut microbiota in maintaining epithelial integrity, immune function, and metabolic homeostasis, we hypothesized that dietary supplementation with elevated levels of antioxidant vitamins (C and E) and organic forms of zinc and selenium could attenuate heat stress-induced dysbiosis [17, 18]. Specifically, we expected that such interventions would help preserve microbial diversity, suppress opportunistic taxa, and support beneficial metabolic functions, particularly short-chain fatty acid production, thereby improving thermotolerance and maintaining performance under thermal stress.

To test this hypothesis, the present study aimed to investigate the impact of chronic heat stress on production parameters such as daily gain and FCR, the composition, diversity, and functional potential of the porcine gut microbiota, and to evaluate whether these adverse effects can be mitigated by micronutrient-enriched diets. A multi-phase experimental design was employed, integrating taxonomic profiling, functional inference, and microbial network analysis to (i) identify microbiome-level signatures of thermal stress, (ii) determine the most responsive microbial taxa and metabolic pathways, and (iii) assess the extent to which dietary interventions could enhance microbial resilience and preserve intestinal homeostasis.

## Results

### Description of the study

**Fig. 1:**
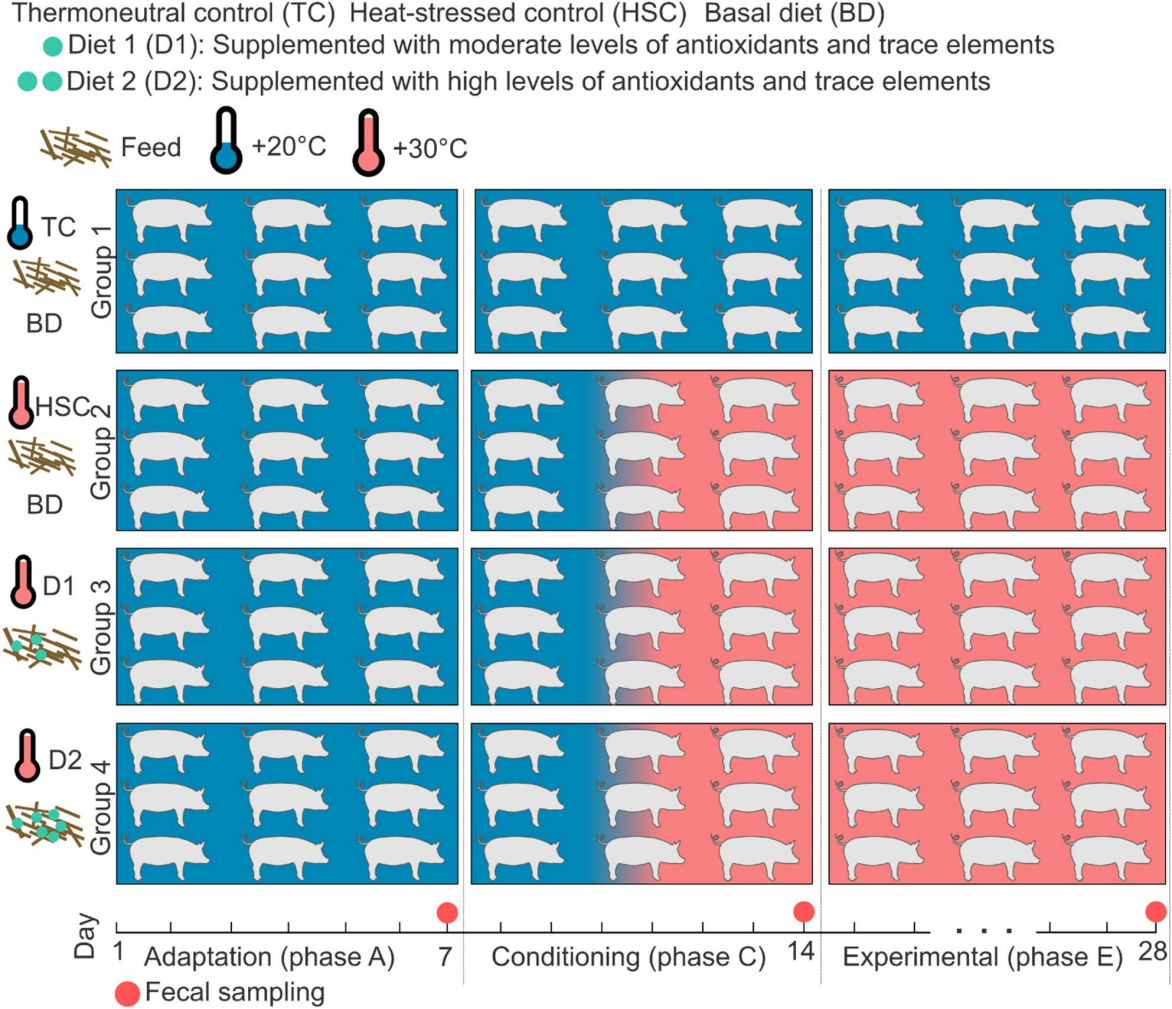
Experimental design and animal allocation**.** A total of 36 DanBred hybrid castrated pigs (barrows) were randomly allocated to four treatment groups (*n* = 9 per group). The study was conducted in three consecutive phases: Adaptation (A) (Days 0–7), Conditioning (C) (Days 8–14), and Experimental (E) (Days 15–28). All pigs were housed under thermoneutral conditions during the adaptation phase and fed a standard basal diet (BD). During the conditioning phase, Group 1 (TC) remained under thermoneutral conditions and continued on the BD, serving as the thermoneutral control. The remaining animals were subjected to a stepwise heat stress protocol, gradually increasing the ambient temperature to 30 °C, and were divided into three groups: Group 2 (HSC): heat-stressed control, fed the BD. Group 3 (D1): heat-stressed, fed Diet 1 (D1), containing moderate supplementation of Vitamin E and C and Se and Zn (in combination). Group 4 (D2): heat-stressed, fed Diet 2 (D2), enriched with higher levels of vitamin E and C, Se and Zn. All animals had *ad libitum* access to feed and water. Fecal sampling was taken on days 7, 14, and 28, as indicated by red dots. The design allowed for the evaluation of functional dietary interventions in mitigating the physiological and microbiological impacts of chronic heat stress

A total of 36 DanBred hybrid pigs were enrolled in this study (Fig. 1). Upon arrival, pigs were individually tagged and randomly assigned to one of four treatment groups, ensuring a balanced initial weight across pens. The animals were housed in two climate-controlled environments: a thermoneutral room (*n* = 9 pigs, 3 pens) and a high-temperature room (*n* = 27 pigs, 9 pens), each pen housed three pigs.

All pigs underwent a 7-day adaptation phase (A; Days 0–7) under thermoneutral conditions. Following this, pigs in the “heat stress room” were gradually exposed to elevated temperatures over a 7-day conditioning phase (C; Days 8–14), reaching and then maintaining a final room temperature of 30 °C. The experimental phase (E; Days 15–28) was conducted under these sustained heat stress conditions.

During the adaptation phase, all animals received a standard corn–soybean basal diet (BD). Through the study, three dietary treatments were evaluated: Basal diet (BD): standard corn-soybean formulation; Diet 1: BD supplemented with moderate levels of vitamin E, vitamin C, organic zinc, and selenium; Diet 2: BD supplemented with high levels of the same antioxidants and trace elements. The thermoneutral control group (TC, *n* = 9) received only the basal diet throughout the entire study. In the “heat stress room”, pigs were randomly assigned to one of three groups (*n* = 9 each): a heat-stressed control group (HSC) fed the BD, and two intervention groups receiving either D1 or D2 starting from the conditioning phase. This design enabled the assessment of nutritional strategies to mitigate the effects of chronic heat stress in pigs.

### Body weight and FCR under heat stress

**Fig. 2:**
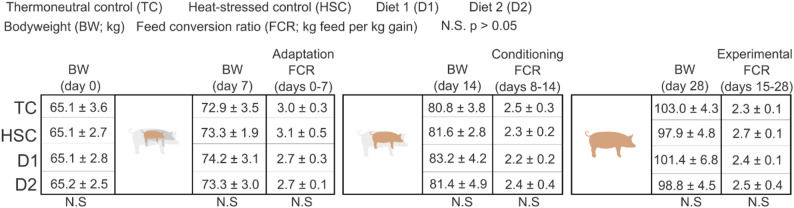
Body weight and feed conversion ratio across phases and treatments**.** Panel set summarizing mean body weight (BW; kg) for sampling points (day 0, day 7, day 14 and day 28) and feed conversion ratio (FCR; kg feed per kg gain) for treatment groups across three experimental phases: Adaptation (A; Days 0–7), Conditioning (C; Days 8–14), and Experimental (E; Days 15–28). Each panel displays a table of group means ± SD for BW and FC. No statistical significance was found (*p* > 0.05). N.S = not significant

Across the three experimental adaptation, conditioning, and experimental phases, body weight (BW) data showed broadly the same trend between treatments, while feed conversion ratio (FCR; kg feed/kg gain) showed the clearest treatment separation during the experimental phase (Fig. 2).

At adaptation (Days 0–7), groups began at comparable body weight (~ 65 kg) and reached ~ 73–74 kg by Day 7 with only minor divergence. Small FCR differences were already evident, TC/HSC ~ 3.0–3.1 vs. D1/D2 ~ 2.7, indicating ~ 11–15% poorer efficiency in controls; however, these were not statistically significant (*p* > 0.05).

In the conditioning phase, relative to TC (2.5), HSC showed an ~ 8% lower FCR (2.3), D1 ~ 12% lower (2.2), and D2 ~ 4% lower (2.4) (not statistically significant, *p* > 0.05).

By the experimental phase (Days 15–28), patterns diverged under sustained heat. TC continued to improve (2.5 → 2.3; -8.0%), whereas HSC worsened markedly (2.3 → 2.7; +17.4%), D1 rise from 2.2 to 2.4 (+ 0.2; +9.0%), and D2 from 2.4 to 2.5 (+ 0.1; +4.2%) (not statistically significant, *p* > 0.05).

### Impact of dietary interventions on gut microbial alpha diversity in heat-stressed pigs

**Fig. 3:**
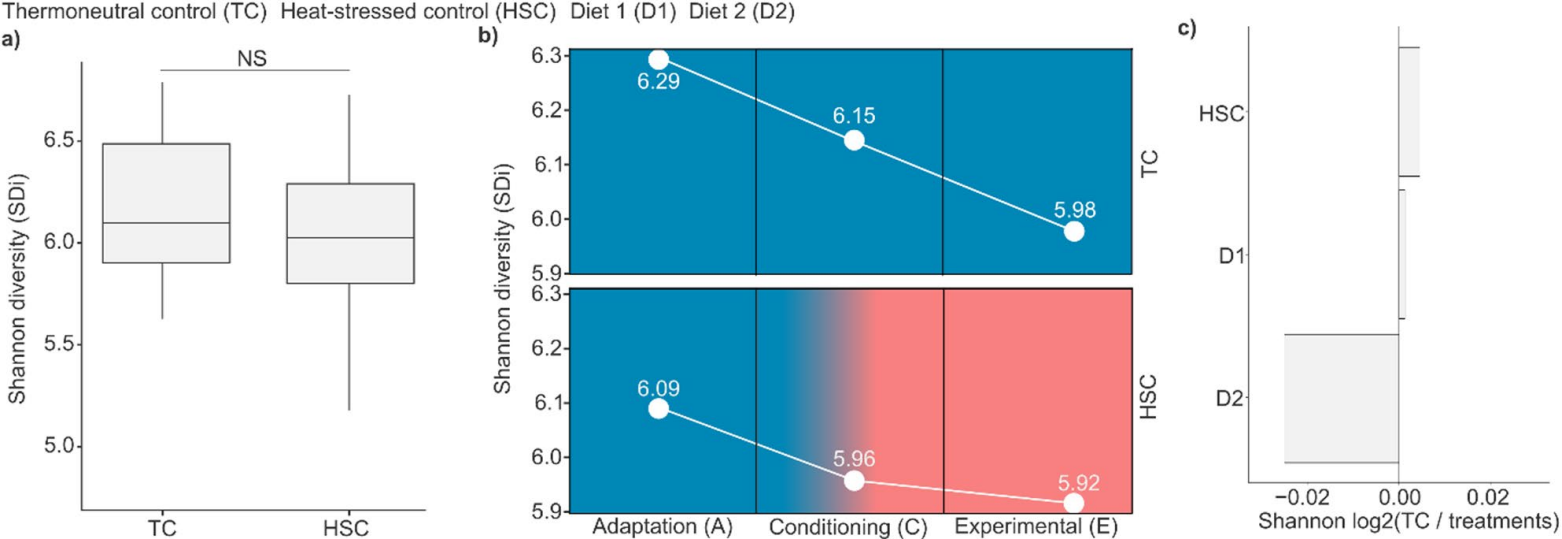
Effects of dietary interventions on gut microbiota’s Shannon indices under heat stress**.** (**a**) Box plots show a comparison of Shannon diversity between thermoneutral control (TC) and heat-stressed control (HSC) groups during the experimental phase. (**b**) Temporal dynamics of Shannon diversity across the adaptation, conditioning, and experimental phases in TC and HSC groups. (**c**) Log₂ fold change of Shannon diversity measured during the experimental phase in heat-stressed groups receiving either the basal diet (HSC), Diet 1 (D1), or Diet 2 (D2), relative to the thermoneutral control (TC). NS = not significant

Changes in gut microbiome alpha diversity (Shannon diversity index; SDi) were examined in pigs, and it was found that, although diversity was slightly lower in the heat-stressed control group compared to the thermoneutral control, the difference was not statistically significant (*p* = 0.37; Fig. 3a).

Tracking Shannon diversity values across the three experimental phases (adaptation; phase A, conditioning; phase C, experimental; phase E) revealed a gradual decline in both TC and HSC groups over time, with the highest diversity observed during the adaptation phase (phase A-SDi-TC: 6.29, phase A-SDi-HSC: 6.09) and the lowest in the experimental phase (phase E-SDi-TC: 5.98, phase E-SDi-HSC: 5.92) (Fig. 3b).

Shannon diversity indices were compared across the experimental groups exclusively during the experimental (E) phase, including a heat-stressed control group receiving the basal diet (HSC) and those receiving distinct dietary interventions (D1 and D2), with the thermoneutral control (TC) serving as the reference (Fig. 3c). By the time animals reached the experimental phase, heat stress was associated with a modest decrease in microbial diversity in both HSC and D1 relative to TC (Shannon diversity, SDi log₂(TC/HSC) = 0.0045; SDi log₂(TC/D1) = 0.0014). In contrast, pigs receiving Diet 2 showed a non-significant increase in Shannon diversity compared with TC (SDi log₂(TC/D2) = -0.025, *p* = 0.16).

### Multilayered reconfiguration of gut microbiota under chronic heat stress by dietary supplementation

**Fig. 4:**
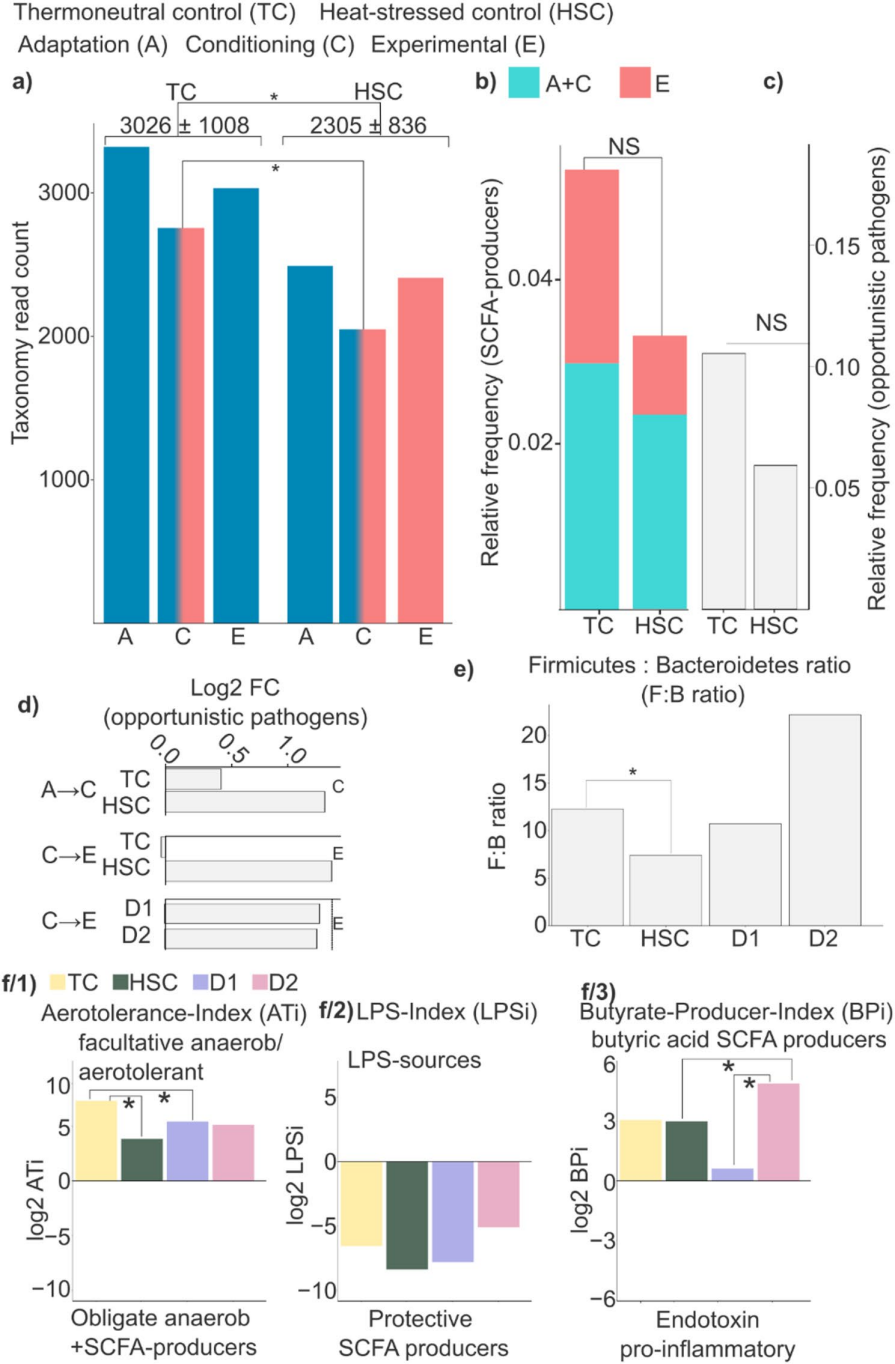
Analysis of taxonomic composition under thermoneutral and heat-stress conditions**.** (**a**) Barplots show the average read counts of GIT microbiota in thermoneutral control (TC) and heat-stressed control (HSC) groups during the experimental phases, such as Adaptation phase (A), Conditioning phase (C), and Experimental phase (E). (**b**) Stacked bars compare the relative abundance of short-chain fatty acid (SCFA)-producing taxa between the thermoneutral control (TC) and heat-stressed control (HSC) groups. Adaptation and conditioning phases were combined (A + C) and compared to the experimental phase (phase E). (**c**) The relative abundance of opportunistic pathogens was compared between the thermoneutral control (TC) and heat-stressed control (HSC) groups. (**d**) Log2 FC values were used to explore the changes in opportunistic pathogen relative frequency across the phases. (**e**) Barplots show the Firmicutes to Bacteroidetes ratio (F: B ratio) in the TC, HSC, D1 and D2 groups. **f**). Functional guild indices (log₂ scale) across treatment groups. **f/1**) Aerotolerance-Index (ATi): positive → shift toward facultative anaerobes/aerotolerant taxa; negative → enrichment of obligate anaerobes/SCFA producers. **f/2**) LPS-Index (LPSi): positive → tilt toward LPS-source taxa; negative → dominance of protective SCFA producers. **f/3**) Butyrate-Producer Index (BPi): positive → relative enrichment of butyrate producers; negative → enrichment of endotoxin-associated, pro-inflammatory taxa. Asterisks indicate significant difference (* *p* < 0.05). NS = not significant

Gastrointestinal tract microbiome profiles were assessed by comparing total read counts between the thermoneutral control and heat-stressed control groups. Average read count analysis revealed significantly higher microbial abundance in the TC group relative to the HSC group (read count TC: 3,026 ± 1,008 vs. HSC: 2,305 ± 836; *p* = 0.014) (Fig. 4a). Although read count distributions across the experimental phases indicated consistently higher microbial abundance in the thermoneutral control group compared to the heat-stressed control group, statistically significant differences emerged only during the conditioning phase. At this stage, the TC group exhibited a significantly elevated microbial load (TC: 2,757 ± 553 vs. HSC: 2,042 ± 686; *p* = 0.042). This effect appears most pronounced during the early phase of thermal exposure, with potential consequences for host physiological adaptation and microbial resilience under sustained heat challenge.

Analysis of the relative abundance of short-chain fatty acid-producing taxa revealed consistently higher proportions in the TC group compared to the HSC group (SCFA relative frequency TC: 0.053, HSC: 0.033); however, these differences did not reach statistical significance (*p* > 0.05) (Fig. 4b). Notably, the observed divergence appears primarily attributable to the experimental phase, as average values for the adaptation and conditioning phases were comparable between groups.

When evaluated across the entire experimental period, without stratification by individual phases (adaptation, conditioning, experimental), the cumulative relative abundance of opportunistic pathogens (OP) (see Supplementary File 1 for the list of taxa and corresponding data) was lower in the HSC group compared to the TC group (OP relative frequency: TC: 0.18 ± 0.03; HSC: 0.10 ± 0.08). However, this difference did not reach statistical significance (*p* > 0.05) (Fig. 4c).

Several notable patterns emerged when examining the relative abundance of OP across the distinct experimental phases (Fig. 4d). During the transition from adaptation to conditioning (A→C), the increase in OP abundance observed in the thermoneutral control group was markedly smaller, approximately one-third compared to the increase recorded in the heat-stressed control group (log₂ A→C-TC: 0.47, log₂ A→C-HSC: 1.36). In the subsequent conditioning-to-experimental transition (C→E), a slight decrease in OP abundance was detected in the TC group, whereas a further, more pronounced increase was observed in the HSC group (log₂ C→E-TC: -0.037 and log₂ C→E-HSC: 1.42). Interestingly, both dietary interventions (D1 and D2) appeared to attenuate the heat stress-associated rise in OP abundance, with D2 exerting a more pronounced mitigating effect than D1. Nevertheless, both supplementation regimens resulted in modest reductions in OP levels relative to the unsupplemented HSC group (for detailed values, see Supplementary File 1).

Chronic heat stress was associated with a marked reduction in the Firmicutes-to-Bacteroidetes (F: B) ratio, indicating a stress-driven compositional shift potentially compromising microbial fermentative capacity and nutrient conversion efficiency (Fig. 4e). Notably, dietary supplementation at the moderate dose partially restored the F: B ratio towards baseline levels, suggesting a stabilizing effect that may enhance microbial resilience and partially offset heat-induced dysbiotic shifts. Intriguingly, high-dose supplementation induced a further elevation of the F: B ratio, exceeding even the values recorded in thermoneutral control animals maintained on the basal diet.

Aerotolerance-Index (ATi, log₂) was highest in TC (7.32) and lowest in HSC (3.84), with D1 (5.43) slightly above HSC, and D2 (5.13) intermediate between HSC/D1 and TC (Fig. 4f**/1**). Because higher ATi denotes a greater contribution of facultative anaerobes/aerotolerant taxa (and lower ATi indicates enrichment of obligate anaerobes and SCFA producers), these patterns indicate that heat stress (HSC) shifted the community toward obligate anaerobes/SCFA producers, while both diets mitigated this shift to a limited extent (D1 ≲ HSC, D2 < TC). Significant pairwise differences are specifically annotated for TC vs. HSC (*p* = 0.011) and TC vs. D1 (*p* = 0.040) (asterisks over the TC-HSC and TC-D1 brackets).

In the case of LPS-Index (LPsi, log₂) all groups fall on the negative side, consistent with dominance of protective SCFA producers over LPS-source taxa (Fig. 4f**/2**). HSC was most negative, D1 (-8.38) was also more negative than TC (-6.56), while D2 (-5.10) was the least negative among heat-stress groups (i.e., comparatively higher LPS-source contribution than HSC/D1, yet still SCFA-leaning relative to zero). No pairwise significance is annotated in this panel (*p* > 0.05; no asterisks).

Butyrate-Producer-Index (BPi, log₂). TC (3.06) showed a moderate BPi, HSC (3.0) was lower, D1 (0.61) the lowest, and D2 (4.89) the highest across groups (Fig. 4f**/3**). Thus, BPi appeared more diet-responsive than heat-responsive: D1 decreased BPi relative to TC, consistent with a shift toward endotoxin-associated, pro-inflammatory taxa under heat stress feed with Diet 1, whereas D2 increased BPi relative to HSC, indicating enrichment of butyrate producers under heat stress feed with Diet 2. Significant pairwise differences are specifically annotated for HSC-D2 (*p* = 0.031) and D1 vs. D2 (*p* = 0.033) (asterisks over the HSC-D2 and D1–D2 brackets).

### Selective genus-level microbial signatures of heat stress in the porcine GIT

**Fig. 5:**
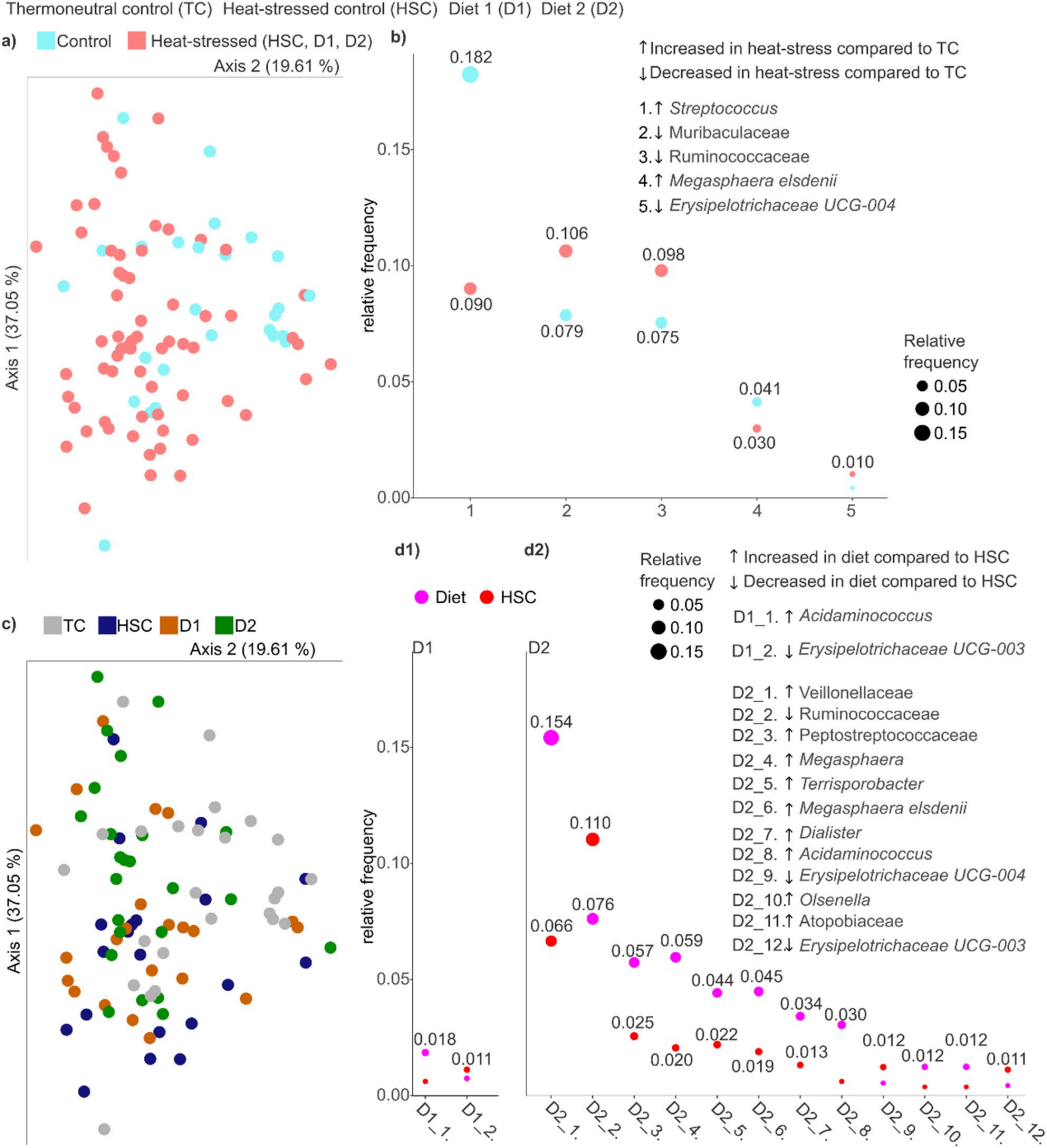
Taxonomic structure and differential abundance dynamics of the porcine gut microbiota in response to chronic heat stress and dietary supplementation. (**a**) Principal coordinates analysis (PCoA) based on Weighted UniFrac dissimilarity of the complete taxonomic dataset in control (TC) and heat-stressed groups (HSC, D1, D2). (**b**) Bubble plot was used to visualize relative frequency of control (TC) and heat-stressed groups (HSC, D1, D2). Relative frequency is proportional with bubble radius and the y-axis, relative abundance threshold > 0.01. (**c**) PCoA based on Weighted UniFrac dissimilarity of the gut microbiota from heat-stressed animals receiving dietary interventions (moderate or high micronutrient supplementation). (**d**) Bubble plot was used to visualize the relative frequency of diet (moderate or high micronutrient supplementation) and HSC groups. Relative frequency is proportional with bubble radius and the y-axis, relative abundance threshold > 0.01

Structural changes in the gut microbiota were assessed by comparing community composition between pigs maintained under thermoneutral conditions and those exposed to heat stress (Fig. 5a). No distinct clustering was observed between the thermoneutral control and heat-stressed groups when clustering was performed based on the complete taxonomic profile, indicating that, under the applied experimental conditions, heat stress did not induce substantial alterations in the overall structure of the porcine gut microbial community.

However, differential abundance analysis identified a subset of five taxa (each exhibiting relative abundances exceeding 0.01%), including two families, two genera, and one species, whose relative abundances were significantly affected by thermal exposure (Supplementary File 2, metacoder analysis) (Fig. 5b). Among these, *Streptococcus* (2.02-fold decrease, *p* = 0.008), and *Megasphaera elsdenii* (1.38-fold decrease, *p* = 0.045) showed significantly lower abundance under heat stress, whereas *Erysipelotrichaceae UCG-004* (2.34-fold increase, *p* = 0.035), Ruminococcaceae (1.29-fold increase, *p* = 0.028), and Muribaculaceae (1.35-fold increase, *p* = 0.022) exhibited significant enrichment indicating that, despite the absence of global compositional restructuring, heat stress induced targeted microbial responses.

The potential influence of dietary interventions on the gut microbiota of heat-stressed pigs was also examined. Consistent with previous findings, no clear clustering was observed, suggesting that dietary supplementation did not induce substantial alterations in the overall microbial community structure (Fig. 5c).

Despite the absence of major shifts in global community structure, differential abundance analysis, applying a relative abundance threshold of > 0.01, enabled the identification of specific taxa that were significantly modulated by dietary supplementation (D1, D2) under heat-stressed conditions. The observed microbial responses were dose-dependent, with distinct taxonomic groups affected by moderate diet: 2 genera and high levels of micronutrient enrichment, encompassing 3 families, 8 genera, and 1 species (Fig. 5d). Moderate supplementation was associated with a statistically significant increase in the relative abundance of the genus *Acidaminococcus* (rf. D1: 0.018, HSC: 0.006), alongside a notable reduction in the family Erysipelotrichaceae (rf. D1: 0.011, HSC: 0.007) (Fig. 5d**/1**). In contrast, high-level supplementation elicited more pronounced effects: the family Veillonellaceae exhibited a significant 2.33-fold increase, while the family Ruminococcaceae decreased by 1.39-fold. Additional taxa exhibiting significant increases under D2 included the family Peptostreptococcaceae (2.05-fold), genera *Megasphaera* (1.33-fold), *Terrisporobacter* (2.00-fold), *Dialister* (2.17-fold), *Acidaminococcus* (1.86-fold), and the species *Megasphaera elsdenii* (1.52-fold) (Fig. 5d**/2**). These findings highlight the capacity of targeted nutritional interventions to modulate distinct microbial taxa within a thermally stressed host environment.

### Genus-level correlations with host stress- and inflammation-related gene expression across experimental groups

**Fig. 6:**
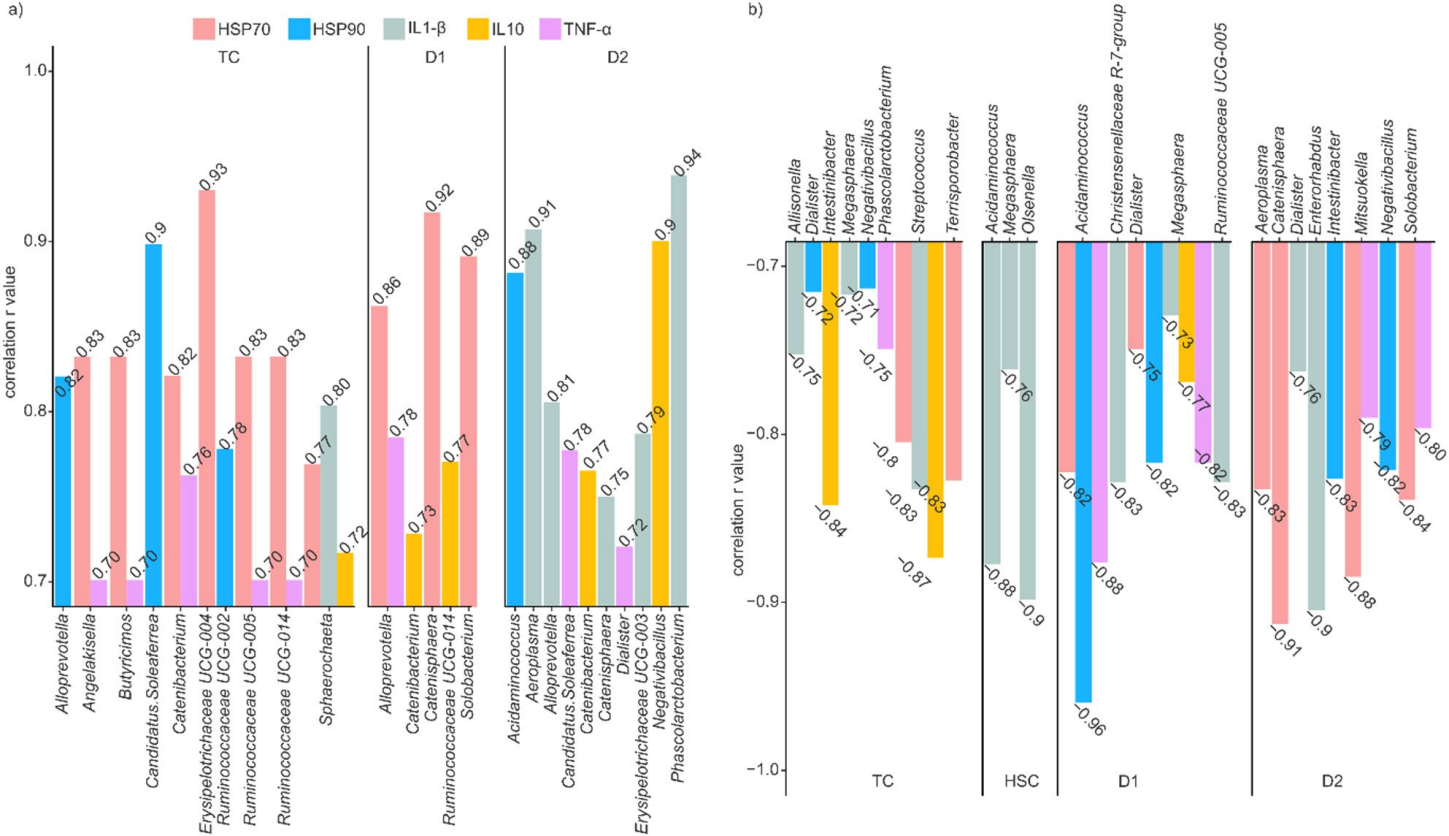
Strong genus-level correlations with qPCR-derived host stress- and inflammatory gene-expression markers across experimental groups. (**a**) Genera showing strong positive correlations and (**b**) genera showing strong negative correlations with qPCR-based expression levels of HSP70, HSP90, IL1B, IL10 and TNFA. Only associations exceeding the predefined strong-effect threshold (*r* > 0.70) are shown across the four study groups (TC, HSC, D1 and D2)

We also examined which genera showed strong correlations with the qPCR-based host gene-expression readouts reported in our companion study [19] and quantified according to the previously described methodology.

Focusing on correlations above the strong-effect threshold (|r| > 0.70), we assessed genus-level associations with HSP70, HSP90, IL-1β, IL-10 and TNF-α across all four experimental groups (TC, HSC, D1 and D2) (Supplementary File 3).

Overall, 25 strong positive **(**Fig. 6a**)** and 24 strong negative **(**Fig. 6b**)** genus-level correlations were identified. Of these, the majority involved HSP70, accounting for 40.0% strong positive correlations and 33.3% strong negative correlations. Notably, no strong positive correlations were detected in the HSC group, whereas strong negative correlations were present in all four groups.

Very strong positive correlations (*r* ≥ 0.90) were restricted to a limited number of taxa: in TC, *Candidatus Soleaferrea* with HSP90 (*r* = 0.90) and *Erysipelotrichaceae UCG-004* with HSP70 (*r* = 0.93); in D1, *Catenisphaera* with HSP70 (*r* = 0.92); and in D2, *Aeroplasma* with IL-1β (*r* = 0.91), *Negativibacillus* with IL-10 (*r* = 0.90), and *Phascolarctobacterium* with IL-1β (*r* = 0.94). In contrast, no very strong negative correlations (*r* ≤ -0.90) were observed in TC, whereas such associations were detected for *Olsenella* with IL-1β in HSC (*r* = -0.90), *Acidaminococcus* with HSP90 in D1 (*r* = -0.96), and *Catenisphaera* with HSP70 (*r* = -0.91) together with *Enterorhabdus* with IL-1β (*r* = -0.90) in D2.

### Taxonomic heterogeneity but conserved core metabolic potential with supplementation-driven functional shifts in the porcine gut microbiome

**Fig. 7:**
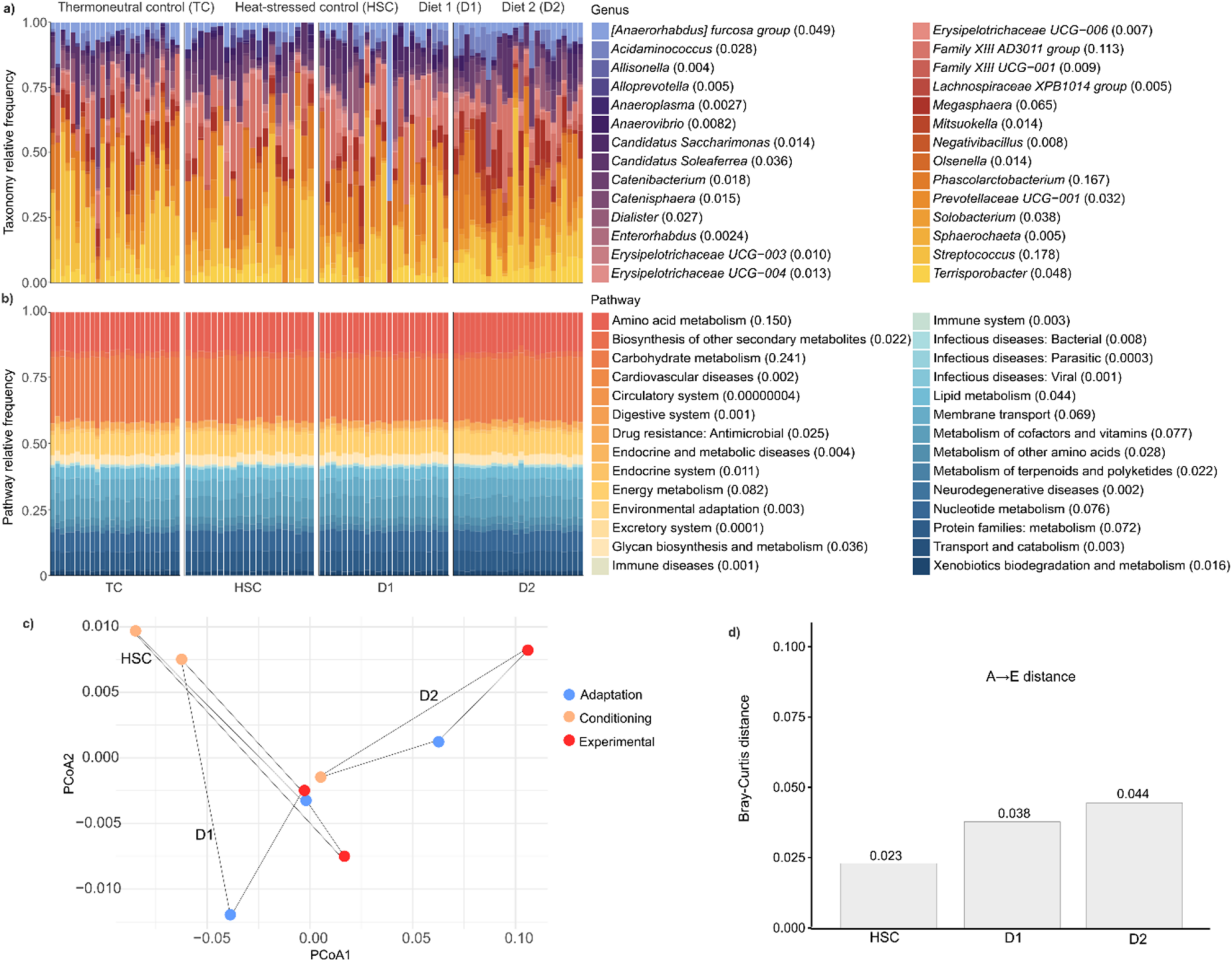
Taxonomic heterogeneity versus functional consistency in the porcine GIT microbiota**.** Stacked bar plots showing the top 28 most abundant genus-level taxonomic composition (**a**) and top 28 most abundant predicted functional pathways (**b**) of the gastrointestinal microbiota across treatment groups: thermoneutral control (TC), heat-stressed control (HSC), and two groups receiving experimental feeds (Diet 1 and Diet 2) under heat stress. (**c**) Results of principal coordinate analysis (PCoA) based on predicted microbial functional metabolic potential inferred from 16 S rRNA gene-derived in silico predictions. Each point represents a sample from one of the three experimental groups (HSC, D1, and D2), and the clustering of points (cluster_1 HSC, cluster_2 D1 and cluster_3 D2) reflects similarities and separation among samples according to their predicted functional metabolic profiles. (**d**) Bray-Curtis dissimilarities between the adaptation and experimental phases based on predicted microbial functional profiles inferred from 16 S rRNA gene–derived in silico predictions. Bar plots represent the distances reflecting the magnitude of shifts in microbiome-associated metabolic potential between phases within the experimental groups

The relationship between taxonomic heterogeneity and predicted metabolic potential of the porcine GIT microbiome was assessed using in silico analyses **(**Fig. 7a, b**)**.

Genus-level taxonomic profiling of the GIT microbiota revealed considerable inter-individual variability, with no clear treatment-specific taxonomic signatures emerging across experimental groups. Emphasis was placed on the 28 most prevalent genera, whose relative abundances were averaged across individual samples within each group. Among these, genera such as *Streptococcus* (relative frequency: 0.178 ± 0.12) and *Megasphaera* (0.065 ± 0.040) consistently ranked among the most dominant taxa across all treatment conditions, with *Streptococcus* exhibiting, on average, a 2.7-fold higher abundance than *Megasphaera* (Fig. 7a).

Functional profiles were also reconstructed to predict community-level metabolic capacities (Fig. 7b). In contrast, functional metagenomic predictions revealed consistent patterns both within and across groups, underscoring the concept of functional redundancy within the microbiome. The most abundantly predicted microbial functions were associated with carbohydrate metabolism (average relative frequency of potential enzyme gene-carrying taxa: 0.241 ± 0.007), amino acid biosynthesis (0.150 ± 0.0028), cofactor and vitamin metabolism (0.077 ± 0.0017), and nucleotide metabolism (0.076 ± 0.0009). In the absence of major perturbations, such as suboptimal husbandry practices, infectious disease outbreaks, systemic physiological disruptions, or metabolic disorders, these findings indicate that core microbial functions remain largely conserved across individual animals, despite pronounced taxonomic heterogeneity. This observed functional stability likely reflects the inherent ecological resilience of the gut microbiota and underscores its pivotal role in optimizing nutrient assimilation within commercial live pig production systems.

We next performed a longitudinal analysis across the experimental groups (HSC, D1, and D2) to quantify the extent of change in the predicted functional potential of the microbiome relative to the corresponding thermoneutral baseline of each group. Thus, functional deviations were assessed in a within-group manner, with each condition referenced to its own HC-derived baseline rather than to a shared external control to examine the temporal dynamics of these inferred functional shifts across groups (Fig. 7c). Notably, samples from the D2 group formed a clearly distinct cluster, separating from the mean profiles of both HSC and D1, consistent with a more pronounced restructuring of microbiome-associated functional potential under this condition.

Within each experimental group, the magnitude of functional reconfiguration was quantified using Bray-Curtis dissimilarities. This within-group analysis captured the extent of shift in predicted microbiome metabolic potential relative to the corresponding group-specific baseline, with larger dissimilarities indicating greater functional restructuring (Fig. 7d). Notably, the HSC group showed the smallest within-group shift, exhibiting the lowest Bray-Curtis distance (Bray-Curtis = 0.023), whereas larger dissimilarities were observed with increasing supplementation, Bray-Curtis distance reaching 0.038 in D1 and 0.044 in D2 indicating that functional restructuring became increasingly more pronounced across the supplemented groups.

### Unique, treatment-specific microbial genera drive divergent functional pathways under heat stress and dietary intervention

**Fig. 8:**
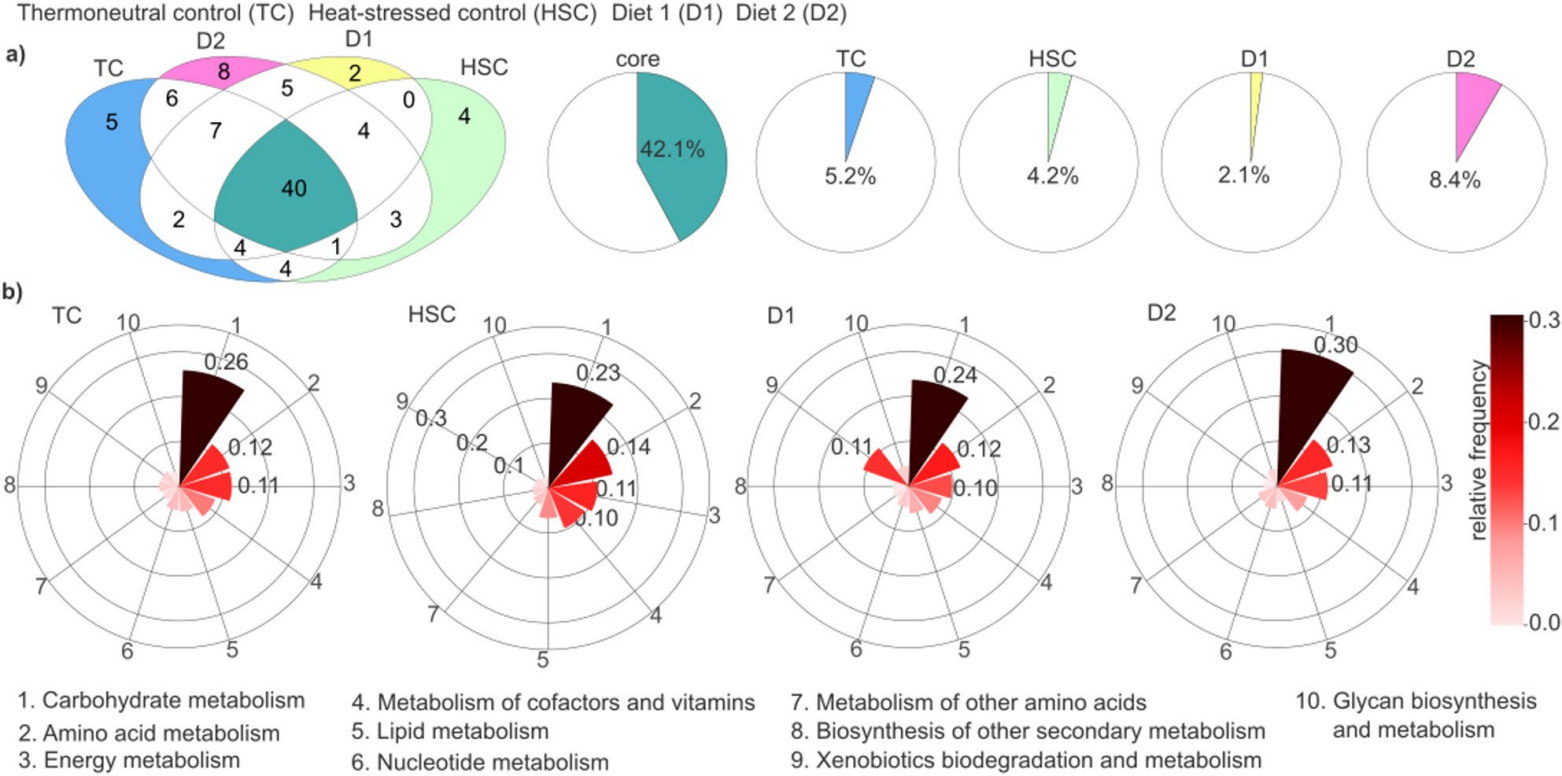
Unique and core genera across experimental groups and their predicted functional contributions to microbial metabolism. (**a**) Venn diagram and pie charts showing the distribution of core and group-specific genera among the four experimental groups: thermoneutral control (TC), heat-stressed control (HSC), and heat-stressed groups receiving experimental diets (D1 and D2). (**b**) Polar plots showing predicted metabolic pathway activity based on in silico-associated gene functions of group-specific genera

Based on genus-level microbial profiles across our four experimental groups, we identified a set of bacterial genera consistently present in all samples, herein defined as the core microbiota. A total of 40 genera, representing 42.1% of all detected genera, were found to be universally present, irrespective of heat stress exposure or dietary intervention (Fig. 8a). This conserved microbial backbone reflects ecological resilience and functional continuity. Among the core taxa, the most dominant genera included *Streptococcus* (mean relative abundance: 0.116 ± 0.126), *Phascolarctobacterium* (0.109 ± 0.045), *Megasphaera* (0.042 ± 0.040), *Terrisporobacter* (0.031 ± 0.022), and *Solobacterium* (0.025 ± 0.016) (Supplementary File 4).

We next identified those core microbiome genera with above-average abundance whose presence and relative frequency were most strongly associated with variation in the five dominant predicted microbial metabolic functions, namely carbohydrate metabolism, amino acid metabolism, energy metabolism, cofactor and vitamin metabolism, and nucleotide metabolism.

Based on canonical correspondence analysis (CCA), *Streptococcus* showed a significant association with the ordination axes (R² = 0.87, envfit permutation test: *p* = 0.001), suggesting its abundance distribution is strongly structured by the measured metabolic functions. In addition, members of the Prevotellaceae family were also associated with the principal microbial metabolic axes. However, this relationship was considerably weaker, indicating a more moderate contribution to the variation observed in the dominant predicted metabolic functions (Supplementary File 5).

In addition to the shared core, we identified group-specific genera uniquely present in individual experimental conditions. The highest number of unique genera was observed in the D2 group (*n* = 8), followed by the TC (*n* = 5), HSC (*n* = 4), and D1 (*n* = 2) groups. The D2 group harbored the fewest, with only 2 unique genera detected.

When examining relative abundance, the unique genera in the D2 group (*Anaerotruncus*, *Desulfovibrio*, *Helicobacter*, *Oscillibacter*, *Roseburia*, *Ruminococcaceae UCG-009*, *Senegalimassilia*, *Sharpea*) collectively accounted for the highest proportion (8.4%) of the total bacterial community, followed by TC (*Dielma*, *Dorea*, *Fusobacterium*, *Phocea*, *Ruminiclostridium 9*) (5.2%) and HSC (*Acetanaerobacterium*, *Lactococcus*, *Mycoplasma*, *Prevotella 1*) (4.2%), while D1 exhibited the lowest relative abundance of unique taxa (*Akkermansia*, *Caproiciproducens*) (2.1%) (Supplementary File 4).

A distinct set of genera, with a combined mean relative abundance of (0.00017 ± 0.00014), was exclusively detected in the TC group, suggesting potential sensitivity to elevated temperatures. In contrast, several genera were absent from TC but present in heat-stressed conditions, indicating microbial adaptation to thermal challenge. The most abundant of these heat-associated genera were *Sharpea* (0.0006 ± 0.0010), *Selenomonas* (0.00058 ± 0.0008), and *Methanobrevibacter* (0.0005 ± 0.00004).

Based on the unique genera identified in each experimental group, we next examined which of the 10 most prevalent biochemical pathway categories detected in the present study, namely (1) carbohydrate metabolism, (2) amino acid metabolism, (3) energy metabolism, (4) metabolism of cofactors and vitamins, (5) lipid metabolism, (6) nucleotide metabolism, (7) metabolism of other amino acids, (8) biosynthesis of other secondary metabolites, (9) xenobiotics biodegradation and metabolism, and (10) glycan biosynthesis and metabolism might be influenced by their relative abundances and associated in silico-predicted gene functions (Fig. 8b).

Among the groups, the highest predicted activity for carbohydrate metabolism was observed in the D2 group (relative frequency: 0.30), followed by the TC group (0.26). Amino acid metabolism consistently ranked as the second most prominent pathway linked to unique genera. Its highest inferred activity was detected in the HSC group (relative frequency: 0.14), followed by the D2 group (0.13), while both the TC and D1 groups exhibited slightly lower values (0.12 each). Several other metabolic pathways exhibited high predicted activity across all groups. Among these, energy metabolism remained consistently prominent, though it did not show significant variation between experimental conditions. In contrast, the metabolism of cofactors and vitamins displayed a downward trend in response to dietary interventions, suggesting potential suppression of these biosynthetic functions. Notably, the D1 treatment appeared to selectively enhance the relative abundance of microbial taxa involved in glycan biosynthesis, indicating a diet-induced shift in structural carbohydrate metabolism.

### Heat stress, along with targeted dietary interventions, drives restructuring of microbial community architecture and alters keystone taxon dynamics

**Fig. 9:**
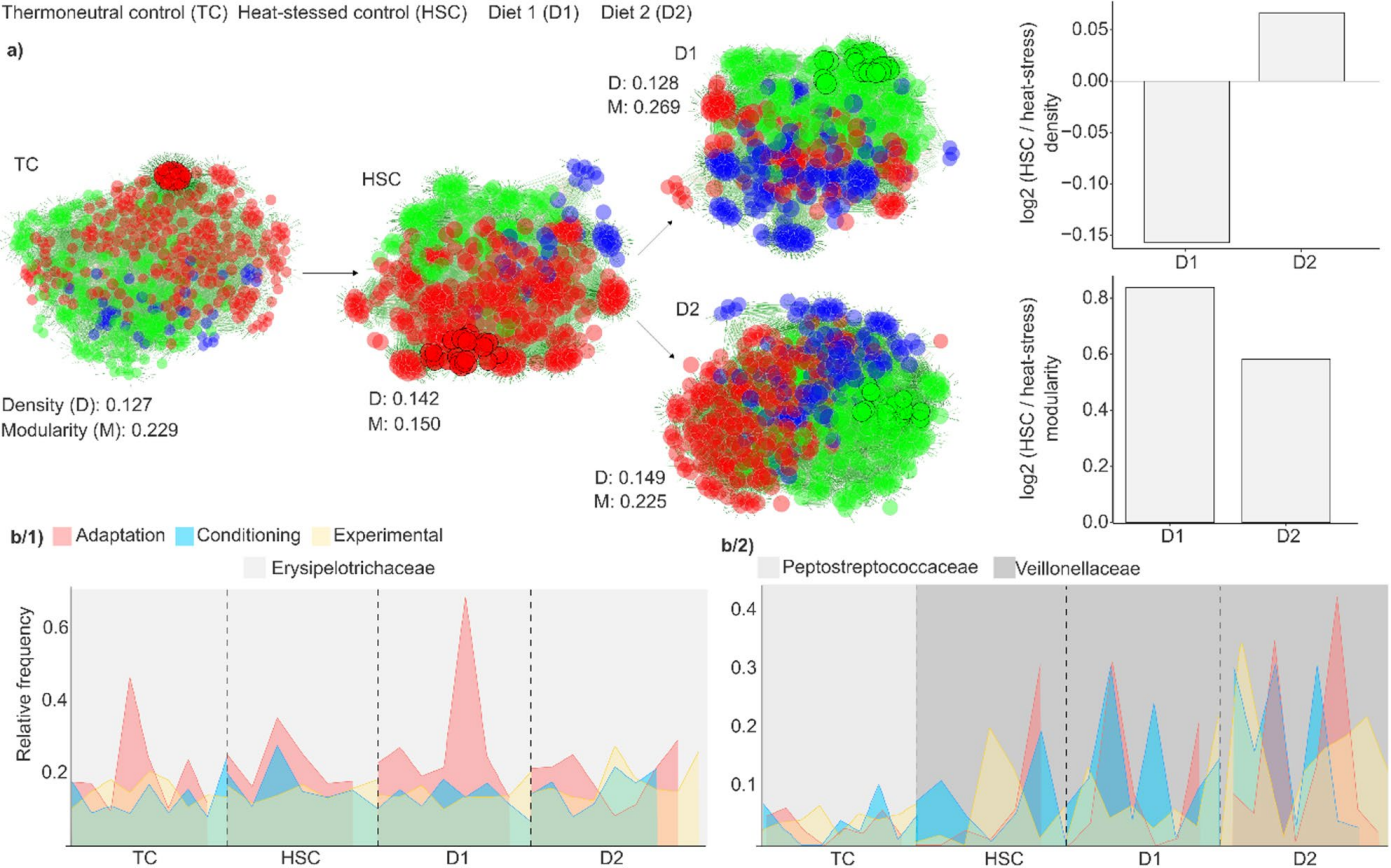
Microbial co-occurrence network architecture and keystone family dynamics under heat stress and dietary intervention. **a**) Co-occurrence network models representing microbial community structure across treatment groups: thermoneutral control (TC), heat-stressed control (HSC), and heat-stressed groups receiving experimental diets (D1 and D2). Node color indicates taxa clusters (green, red, blue), and edge density reflects interaction strength. Log2 fold change in network density and modularity relative to the heat-stressed control in response to dietary supplementation. **b/1**) Relative abundance dynamics of Erysipelotrichaceae, a core keystone family consistently present across all treatments. **b/2**) Distribution of condition-specific keystone families. Peptostreptococcaceae was exclusively detected in the TC group and remained relatively stable across phases. In contrast, Veillonellaceae was only present in the heat-stressed groups (HSC, D1, D2), with differential trends

To assess the impact of heat stress on microbial ecosystem organization, and to determine whether dietary interventions could modulate these effects, we analyzed the gut microbiota co-occurrence network architecture across experimental groups (Fig. 9a).

Under environmental stress, microbial communities may reorganize their interaction networks in different ways. One possible response is an increase in network density, reflecting tighter metabolic interdependence and enhanced cross-feeding among taxa, which may buffer functional performance and confer short-term resistance to disturbance. Heat stress led to a marked reduction in network modularity, with a 1.52-fold decrease compared to the thermoneutral control (TC: 0.229 vs. HSC: 0.150), suggesting a shift toward less structurally compartmentalized microbial communities and a reduction in ecological niche separation under elevated temperature. In contrast, network density increased slightly (TC: 0.127 vs. HSC: 0.142), potentially reflecting a compensatory rise in overall connectivity aimed at preserving functional stability despite the loss of modular structure.

In ecological interaction networks, higher modularity is generally associated with greater resilience. A modular network is organized into semi-independent clusters of interacting taxa, which can buffer the community against disturbance. To evaluate the modulatory potential of dietary strategies under heat stress conditions, we assessed the effects of Diet 1 and Diet 2. D1 notably enhanced modularity, resulting in a 1.79-fold increase relative to the heat-stressed control (log2[HSC/D1] modularity = 0.838). D2 also promoted modularity, albeit to a slightly lesser extent (1.50-fold; log2[HSC/D2] modularity = 0.583), indicating that both diets supported a reorganization of the microbial network toward increased structural compartmentalization.

Network density responded differently to the two dietary interventions. D1 induced an 11% decrease in density (log2[HSC/D1] = − 0.157). In contrast, D2 increased density by 5% (log2[HSC/D2] = 0.067), pointing to a broader increase in both modular organization and network-level connectivity. Collectively, these results imply that Diet 1 and Diet 2 modulate the gut microbial network architecture via distinct mechanisms. D1 by reinforcing modular structure while reducing overall connectivity, and D2 by simultaneously increasing both modularity and interaction density.

We analyzed keystone bacterial families across the experimental groups, identifying them based on co-occurrence network metrics. Among these, Erysipelotrichaceae emerged as a core keystone family consistently present across all conditions, regardless of heat stress or dietary intervention (Fig. 9b**/1**). To further explore its ecological role, we tracked the relative abundance of Erysipelotrichaceae across the adaptation, conditioning, and experimental phases within each treatment group. Notably, its abundance peaked during the adaptation phase in all groups. In the TC group, the mean relative abundance of Erysipelotrichaceae was highest during adaptation (relativefrequency: 0.20), compared to conditioning (0.14) and experimental phases (0.15). Similar patterns were observed in HSC (adaptation: 0.23; conditioning: 0.16; experimental: 0.15), D1 (0.28; 0.13; 0.15), and D2 (0.19; 0.16; 0.18). The highest overall levels were seen in the TC and D1 groups, suggesting that Erysipelotrichaceae may contribute to early-stage microbial stabilization or stress priming. These findings support its role as a robust, functionally relevant member of the porcine core microbiota across environmental and nutritional contexts. In addition to core keystone families, we identified condition-specific keystone taxa restricted to particular experimental settings. Peptostreptococcaceae was detected exclusively in the TC group, while Veillonellaceae was consistently present in all heat-stressed groups (HSC, D1, and D2), but absent in the thermoneutral condition (Fig. 9b**/2**).

Distribution profiles revealed that Peptostreptococcaceae maintained stable abundance across experimental phases in the TC group, with a modest increase during the experimental phase (0.046). In contrast, Veillonellaceae exhibited greater temporal fluctuation. While its relative abundance declined in D1 compared to HSC, it increased in D2, suggesting differential responsiveness to dietary supplementation. These patterns imply that Peptostreptococcaceae may contribute to microbial stability under thermoneutral conditions, whereas Veillonellaceae likely plays a role in microbiota restructuring during heat stress, modulated by diet-specific effects.

## Discussion

In 2023, Hungary accounted for approximately 1.8% of the European Union’s total pork production (EU-28), with an annual output of 420,000 tonnes and a pig population ranging between 2.5 and 2.7 million head, placing the country between 11th and 13th among EU Member States in terms of production volume [20–22].

Climate change poses growing risks to the Hungarian pig sector, with increasingly frequent heat waves and erratic precipitation patterns exacerbating both physiological and infrastructural stress [23, 24]. Pigs are particularly susceptible to thermal stress due to inefficient thermoregulation and high metabolic heat production, making even moderate temperature increases detrimental to immune function, gut integrity, and microbiota-host interactions [5, 8]. In addition to microbial disruptions, heat stress adversely affects production of swine (e.g. feed intake, feed conversion ratio, and reproductive performance). Finishing pigs may show up to a 15% decline in feed intake, while sow fertility can drop by 5–10% during peak heat periods [6, 24, 25].

This study examines the impact of chronic heat stress, both as an isolated environmental stressor and in conjunction with targeted dietary supplementation on the gastrointestinal microbiota of DanBred hybrids, by simulating relevant thermal stress either alone or alongside micronutrient-enriched nutritional interventions aimed at mitigating climate-induced physiological strain.

A total of 36 pigs (barrows) were housed in pens (3 animals/pen) in climate-controlled chambers, enabling high-resolution, individual-level monitoring under standardized conditions. Body weight (BW) gains were maintained across treatments, but sustained heat imposed a clear efficiency cost on the basal diet, evident as a higher feed conversion ratio (FCR) in the heat-stressed group (HSC). This observation aligns with previous studies investigating the impact of heat stress on livestock performance [26–28]. Both supplemented diets (D1, D2) partially mitigated this penalty, most consistently D2.

During adaptation, body weights tracked in parallel across groups, yet a modest FCR advantage already emerged in the supplemented animals. During conditioning, FCR briefly improved even in the heat-stressed control, moderate antioxidant/trace-element supplementation appeared most effective at this stage, whereas the higher dose conferred no additional benefit, consistent with a mild U-shaped (hormetic) response pattern [29, 30]. Under sustained heat, FCR deteriorated in unsupplemented controls, whereas supplementation partially mitigated these effects, with higher doses providing greater stability over time. Heat stress is known to increase oxidative and physiological strain, disrupt intestinal barrier integrity, and alter inflammatory signaling, all of which can compromise nutrient utilization [5, 6, 8]. Although intestinal barrier integrity and inflammatory responses were not measured in the present study, other work has shown that heat stress can compromise these factors and. nutritional interventions, like antioxidants, trace elements, or trehalose, can attenuate these effects and support microbial and physiological homeostasis [31, 32]. Therefore, vitamins E and C with selenium and zinc may have contributed to maintaining feed efficiency under heat stress, consistent with a phase-dependent, hormetic pattern in which moderate doses are beneficial early and higher doses stabilize responses under prolonged stress [16, 19, 29, 33, 34].

Consistent with previous reports across mammalian models, our findings confirm that heat stress exerts a detrimental effect on gut microbial diversity in pigs [7, 35, 36]. Reduced alpha diversity is frequently associated with dysbiosis, heightened intestinal inflammation, impaired nutrient absorption, and compromised immune function, all of which may contribute to diminished production efficiency [37, 38].

Both the thermoneutral and heat-stressed control groups exhibited a similar relative reduction in Shannon diversity over the course of the trial (SDi: ~2.2% and ~ 2.1%, respectively). One plausible explanation for the decline in diversity across the adaptation- conditioning- experimental trajectory is the combined ecological impact of dietary transition and heat stress. The feed change following the adaptation phase likely imposed strong substrate-driven selection on the gut microbiome, favoring taxa capable of efficiently exploiting the new nutritional environment while reducing the abundance of less competitive community members [39]. Furthermore, during the adaptation phase, before the onset of heat stress, baseline diversity values were already approximately 4% higher in the TC group compared to the HSC group, indicating that the initial compositional complexity of the two groups was not fully equivalent. This discrepancy may be partially attributed to a range of factors, including natural inter-individual variability and differential responses to the stress associated with relocation, social separation, and experimental enrolment [40, 41]. Such heterogeneity is reflective of real-world livestock systems, where animals vary in microbiota composition and stress sensitivity even under standardized management.

By the end of the pilot, during the experimental phase, the magnitude of Shannon diversity change in both HSC and D1 remained broadly comparable to that observed in the thermoneutral control, whereas D2 exhibited a marked increase relative to TC. Although the mechanisms underlying this pattern were not directly resolved in the present study, several non-mutually exclusive explanations may account for this observation. One possibility is that the higher-dose supplementation exerted a broader ecological influence on the gut environment, expanding the range of metabolically permissive niches and thereby allowing a wider spectrum of microbial taxa to persist under heat stress. In contrast, the lower-dose intervention may have been insufficient to counterbalance stress-associated ecological filtering at the community level, resulting in little net change, or even a slight reduction in diversity relative to the control. Importantly, the diversity increase observed in D2 should not necessarily be interpreted as an unequivocally beneficial outcome, as it may also reflect a more dynamic phase of community reassembly under the combined pressures of dietary intervention and thermal stress. Similar patterns have been reported in previous studies, where heat stress reduced alpha-diversity indices, while dietary supplementation restored microbial diversity to levels comparable to those observed in thermoneutral control groups [10, 42].

This interpretation was consistent with the persistently elevated Firmicutes: Bacteroidota (F: B) ratio observed in D2 across the full experimental trajectory, including the adaptation, conditioning, and experimental phases. When considered together with the reduced Shannon diversity detected at the experimental stage, this pattern suggests that Diet 2 may have promoted a more strongly selected and less even microbial community structure, characterized by disproportionate expansion of Firmicutes relative to Bacteroidota.

Importantly, D2 was also the group that separated most clearly from the others based on predicted metabolic potential, indicating that compositional changes were accompanied by the most pronounced shift in microbiome-associated functional organization This pattern could reflect broader niche availability or reduced competitive exclusion under the combined influence of supplementation and heat stress, thereby allowing a wider range of taxa to persist. Because these interpretations are based on correlative patterns and predicted functional profiles, further targeted studies will be required to clarify the mechanistic basis and physiological implications of this community state.

Comparison of the thermoneutral and heat-stressed control groups (both receiving the basal diet) revealed peak microbial abundance during the initial adaptation phase, followed by a decline during conditioning. This reduction likely reflects cumulative psychosocial stress induced by regrouping, relocation to unfamiliar pens, and repeated handling and sampling, representing a non-thermal, non-nutritional stress response during environmental adaptation [43], an anticipated yet relevant confounder under the given experimental conditions.

In the absence of significant changes in body weight or feed conversion efficiency, both tightly linked to gut microbiota function, the observed microbial shifts likely reflect a selective reduction in taxonomic diversity buffered by functional redundancy. This suggests that core metabolic functions are preserved despite compositional restructuring, consistent with the concept of a resilient, functionally robust microbiome capable of adapting to environmental or physiological stress [44]. These findings could indicate that under sustained abiotic stress, the microbiota probably undergoes a strategic contraction, removing non-essential, functionally redundant taxa while maintaining key microbial populations critical for host metabolic stability [45].

In a previous publication from the same pilot experiment [19], physiological responses were assessed through nutrient digestibility, plasma metabolites, and intestinal gene expression of stress and immune markers (HSP70, HSP90, IL-1β, IL-10, TNF-α). Elevated HSP70 expression in heat-exposed pigs confirmed activation of the cellular heat shock response and verified effective thermal challenge. Heat stress did not markedly impair nutrient digestibility, plasma metabolites, or consistently upregulate pro-inflammatory cytokines. However, antioxidant supplementation improved nutrient retention, increased IL-10 expression, reduced TNF-α expression, and, at the highest supplementation level, lowered HSP70 mRNA abundance relative to unsupplemented heat-stressed pigs [19].

The correlation architecture observed here indicates that microbiota-host interactions linked to stress and inflammatory signaling were strongly conditioned by physiological context. This interpretation is biologically plausible, as intestinal adaptation to heat stress is closely tied to barrier dysfunction, microbial restructuring, and coordinated regulation of heat-shock and cytokine-related pathways in pigs [7]. Because the host-side variables analysed here were qPCR-derived relative transcript abundances of HSP70, HSP90, IL1B, IL10, and TNFA, rather than protein concentrations, these associations should be interpreted primarily as relationships with the host transcriptional response landscape, not as direct evidence of protein-level activity.

Within this framework, the HSC group is especially informative, as these animals experienced heat stress in the absence of experimental dietary support and therefore likely represent the least buffered physiological state in the study. The absence of strong positive genus-host correlations in this group may thus reflect a partial uncoupling of coordinated microbiota-host transcriptional relationships under uncompensated thermal challenge.

At the genus level, several of the observed associations are biologically credible in light of prior literature, although the strength of mechanistic support differs substantially among taxa. *Phascolarctobacterium*, which showed one of the strongest positive correlations in D2, is among the best supported functionally. Members of this genus are succinate-utilizing, propionate-producing anaerobes, and recent mechanistic work showed that *Phascolarctobacterium succinatutens*-derived propionate can alleviate colonic inflammation by inhibiting TLR4 signaling [46]. By contrast, Erysipelotrichaceae-related taxa are more ambiguous. This family has repeatedly been linked to inflammatory and metabolic disorders, yet review-level synthesis also emphasizes substantial within-family heterogeneity and variable immunogenicity [47].

A significant reduction in the Firmicutes-to-Bacteroidetesratio was observed under chronic heat stress, with potential consequences for fermentative capacity and energy metabolism [48]. Notably, dietary supplementation modulated this effect in a dose-dependent manner: moderate doses partially restored the F: B ratio towards baseline, while high-dose supplementation elevated it above thermoneutral control levels. Heat stress in livestock animals has been associated with significant shifts in the F: B ratio, generally involving a decrease in *Bacteroidetes* and a concomitant increase in *Firmicutes* [36, 48, 49]. This reductive trajectory also extended to short-chain fatty acid-producing taxa. Compared to thermoneutral controls, heat-stressed pigs displayed a marked decline in SCFA-producing taxa, particularly during the experimental phase. These findings might offer a nuanced perspective on the concept of function-preserving diversity reduction, suggesting that while certain core metabolic functions may persist, specific beneficial activities, such as SCFA biosynthesis, may nonetheless be impaired under prolonged environmental stress. Comparable microbial shifts have been reported in other heat-stress models, where elevated temperature compromised the abundance of SCFA-producers, reduced fermentative capacity and lowered SCFA concentrations [35, 36, 46, 50, 51].

While our predicted functional profiles indicate that core metabolic capacities remain relatively stable across groups, this stability likely arises from the presence of multiple microbial taxa with overlapping biochemical capabilities. Within such a functionally redundant framework, however, shifts in the relative abundance of specific taxa, particularly metabolically influential groups such as butyrate-producing bacteria may still have important ecological and physiological implications, even when the overall functional repertoire of the community appears preserved. In this sense, functional redundancy may buffer ecosystem-level metabolic capacity, whereas taxon-level changes may influence the efficiency, regulation, or spatial organization of these metabolic processes within the gut environment [52].

Furthermore, the elevated Shannon diversity observed in the high-supplementation group (D2) relative to the thermoneutral control may represent a double-edged ecological outcome. While increased diversity can broaden the functional repertoire and adaptive capacity of the microbiome under environmental stress, it may also reflect ongoing community restructuring or niche expansion triggered by nutritional perturbation. Whether this increase ultimately contributes to greater ecological stability or instead reflects a more dynamic community state remains unclear.

Our data indicate that chronic heat stress amplifies the relative abundance of opportunistic pathogens, particularly during the later phases of exposure, whereas thermoneutral conditions exerted a stabilizing effect on this parameter. Importantly, both of our dietary interventions demonstrated a protective role by partially mitigating pathogen overgrowth under heat stress, with the higher-level supplementation displaying slightly better efficacy. In large-scale pig production, dietary supplementation with micronutrients and antioxidants has been shown to significantly influence the gastrointestinal microbiome of piglets, particularly affecting the prevalence of potential pathogens [53]. Research also indicates that high levels of certain micronutrients, such as zinc oxide (ZnO), can modulate gut microbial composition and metabolic profiles, thereby impacting piglet health and pathogen dynamics [54, 55]. Furthermore, a dose-response study on weaned piglets fed varying zinc levels demonstrated that the highest dietary zinc inclusion altered the gut microbiota by increasing beneficial bacterial groups involved in fiber fermentation and short-chain fatty acid production, such as *Clostridium sensu stricto* and Prevotellaceae, while decreasing the abundance of potentially pathogenic genera like *Methanobrevibacter*, *Treponema*, and *Peptococcus* [54].

Although in this study pigs antioxidant states were not measured, supplementation with antioxidants and micronutrients such as Cu, Se, and Zn has been associated with improved antioxidant status in piglets, as reflected by elevated activities of enzymes like superoxide dismutase and catalase, alongside reduced oxidative stress markers such as malondialdehyde [56, 57]. These improvements in redox balance may indirectly support the gut microbiome by mitigating oxidative damage and inflammation, thereby reducing factors that would otherwise favor pathogen colonization [58, 59].

Under chronic heat stress, the Aerotolerance Index (ATi) / LPS-Index (LPsi) / Butyrate-Producer-Index (BPi) profiles indicate coherent, directionally consistent shifts. The Aerotolerance Index ranked TC > D1 > D2 > HSC, suggesting that heat stress shifted the microbiome away from obligate anaerobes, including many SCFA producers, toward facultative anaerobic and other oxygen-tolerant taxa. Relative to TC, this shift was most pronounced in HSC, partially normalized in D1, and remained intermediate in D2. Accordingly, a higher ATi reflects greater representation of aerotolerant community members and reduced obligate anaerobe dominance.

This pattern aligns with the established physiological consequences of chronic heat load-reduced feed intake [60], diminished splanchnic perfusion, and lower mucosal oxygenation [61], which collectively drive a more hypoxic luminal milieu and thereby favor obligate anaerobic guilds. Within this framework, D1 appears to mitigate the aerotolerance decline more effectively than D2. Notably, the LPS-index proved to be negative in all groups, indicating an overall predominance of SCFA-leaning guilds over LPS sources with the heat-stressed control being the most negative, D1 also below TC, and D2 the least negative among heat-stressed groups yet still on the SCFA side. Although these LPsi differences were not annotated as significant and should be interpreted as directional, an SCFA-dominant profile is typically associated with a more barrier-protective, less endotoxin-stimulatory milieu. In principle, such communities, particularly when butyrate producers are present, support epithelial integrity, lower permeability, and anti-inflammatory signaling [62], which may be advantageous under heat stress, where splanchnic hypoperfusion and leakiness are concerns. In this context, D2 having the least negative LPsi among heat-stressed groups, while simultaneously increasing BPi, yields a combination (SCFA-leaning + higher butyrate capacity) that is more likely beneficial than harmful, even if the relative contribution of LPS-source taxa is marginally higher than in HSC/D1.

While no overarching shifts in global community architecture were detected between thermoneutral and heat-stressed piglets, suggesting that heat stress per se may not induce broad-scale remodeling of the gut microbiome, differential abundance analyses identified a discrete subset of taxa that exhibited significant compositional changes. Under heat stress, the increase in *Streptococcus*, an abundant genus involved in carbohydrate metabolism, may reflect microbial adaptation to altered nutrient digestion dynamics under thermal challenge [63]. The literature suggests that *Streptococcus* may be particularly responsive to heat stress; however, the direction of this response is not uniform. In poultry, heat exposure has mostly been associated with increased abundance, whereas porcine studies report both decreases and temperature-dependent increases [36, 48, 64]. Furthermore, late-gestational heat stress in sows has been linked to reduced *Streptococcus* levels [65]. Such variability underscores the complexity of microbial adaptation to thermal stress and highlights the influence of host-specific and environmental factors.

Furthermore, the strong association of *Streptococcus* with the dominant predicted metabolic functions may reflect not only its high relative abundance within the porcine GIT microbiome, but also its central ecological position as a metabolically active and broadly connected genus. As one of the most abundant core taxa, *Streptococcus* is likely to make a disproportionate contribution to community-level functional predictions, particularly in pathways related to carbohydrate utilization, amino acid metabolism, energy metabolism, cofactor and vitamin metabolism, and nucleotide metabolism. This finding is biologically plausible given the well-established saccharolytic capacity of many *Streptococcus* spp. and their potential role in shaping downstream cross-feeding interactions within the gut ecosystem [66, 67]. In this context, the observed CCA pattern does not necessarily imply that *Streptococcus* uniquely drives these functions, but rather that variation in its abundance may serve as a sensitive indicator of broader microbiome-level metabolic organization.

The enrichment of *Megasphaera elsdenii*, a lactate-utilizing, SCFA-producing species suggests compensatory microbial support for gut energy homeostasis and epithelial barrier maintenance [68]. The observed increase in Eggerthellaceae, known for its role in polyphenol and host-derived compound metabolism, may also be linked to heat-induced shifts in host-microbe metabolic interactions, potentially modulated by antioxidant-rich dietary components [69]. Conversely, Muribaculaceae, Ruminococcaceae, and Erysipelotrichaceae were more abundant under thermoneutral conditions. These taxa are typically involved in fiber degradation, SCFA production, and gut health maintenance [70, 71]; thus, their reduction may indicate impaired fiber fermentation capacity, consistent with previous reports of heat-induced disruptions in microbial fermentation efficiency [7].

While our dietary interventions in heat-stressed piglets did not induce significant compositional shifts, consistent with prior observations of microbiome resilience under nutritional modulation [72, 73], they triggered distinct, taxon-specific responses. Notably, supplementation enriched Veillonellaceae, which includes SCFA-producing lactate utilizers both critical for maintaining intestinal barrier integrity and modulating inflammation [74–76].

In parallel, increases in Peptostreptococcaceae and *Dialister* align with reports implicating these taxa in gut homeostasis and anti-inflammatory processes [77]. The enrichment of *Terrisporobacter* and *Acidaminococcus*, both involved in amino acid metabolism and fermentation, suggests enhanced microbial metabolic versatility that may support nutrient absorption, nitrogen balance, and immune modulation under heat stress [78, 79]. In contrast, Ruminococcaceae, a core butyrate-producing, fiber-degrading family, showed modest declines under supplementation, potentially reflecting adaptive microbial reorganization that favors taxa more capable of rapidly responding to host metabolic demands during thermal stress [62].

Group-specific taxa and functions were observed: thermoneutral controls harbored *Dielma*,* Dorea*, and *Fusobacterium*, while the high-dose D2 group showed the greatest number of unique genera, including *Anaerotruncus*,* Desulfovibrio*,* Helicobacter*,* Oscillibacter*, and *Roseburia*, linked to fiber degradation, mucosal interaction, and immunomodulation [80–83]. Carbohydrate metabolism dominated across groups, with the highest levels in D2, whereas amino acid metabolism peaked in heat-stressed controls, reflecting adaptive microbial responses to increased proteolytic activity. Metabolism of cofactors and vitamins decreased with supplementation, potentially due to a reduced microbial demand for endogenous biosynthesis as a result of exogenous micronutrient intake. These results support previous reports that heat stress induces selective taxonomic shifts rather than wholesale community restructuring [10, 47].

Based on our observations, the TC network appeared to represent a moderate density with relatively high modularity. A more rigorous interpretation is that the TC network already occupied a structurally balanced state, with moderate density (0.127) and relatively high modularity (0.229), a range broadly consistent with microbial co-occurrence networks in other stressed and non-stressed systems, where moderate connectivity is often interpreted as supporting efficient interaction without excessive fragility, and modularity in the ~ 0.2–0.3 range is commonly linked to compartmentalization and disturbance buffering [84].

Reviews of microbial network ecology and empirical studies across environmental microbiomes similarly emphasize that modular organization can enhance resilience by limiting perturbation spread across the whole community [85]. Against this background, heat stress displaced the network from this balanced architecture, most notably through a 1.52-fold reduction in modularity, accompanied by only a 1.12-fold increase in density. Ecologically, this pattern might suggests weaker structural compartmentalization, reduced niche separation, and diminished buffering capacity at the module level, together with a modest compensatory tightening of interactions among the remaining taxa. Thus, in our system, heat stress appeared to have promoted a shift toward a less compartmentalized but slightly more interconnected network configuration.

The dietary responses were particularly informative because they pointed to two distinct stabilization modes rather than a single common recovery pattern. In D1, density remained essentially unchanged relative to TC, whereas modularity increased, corresponding to a 1.17-fold increase over TC. This pattern is most consistent with a modularity-driven recovery, in which resilience is restored primarily through stronger structural compartmentalization without substantial expansion of overall connectivity.

By contrast, D2 displayed a different response profile. Here, modularity remained close to the TC baseline, indicating little net change in compartmentalization, whereas density increased, corresponding to a 1.17-fold increase relative to TC. Ecologically, such a configuration may enhance metabolic interdependence and cross-feeding among taxa, thereby supporting functional buffering through tighter community-level integration.

Taken together, these findings suggest that the two dietary interventions may have mitigated the structural effects of heat stress through alternative ecological routes: D1 by reinforcing modular resilience, and D2 by strengthening interaction-based connectivity while maintaining baseline compartmentalization which distinction might be important, as it implies that microbiome stability under stress may be achieved not through a single optimal topology, but through different network-level solutions with potentially different trade-offs between resilience, robustness, and perturbation propagation. Animal studies showed that elevated temperature disrupts gut microbial community structure. In rodents, heat exposure altered microbiome composition and reduced stability, shifting key taxa under thermal challenge [86]. Similar structural perturbations have been reported in broilers and pigs, where heat stress modified major bacterial groups, reduced beneficial taxa, and promoted opportunistic populations [35, 59, 87]. These findings could further support the interpretation that elevated temperature undermines microbial network compartmentalization, while altered connectivity patterns may reflect adaptive reorganization under stress.

Our keystone taxa analysis further contributes to the field by identifying Erysipelotrichaceae as a consistent core keystone family across all conditions, peaking during the adaptation phase. This supports previous reports [88] that highlight Erysipelotrichaceae*’s* role in gut microbial stabilization and host immune modulation. The condition-specific keystone taxa we identified Peptostreptococcaceae exclusive to thermoneutral controls and Veillonellaceae specific to heat-stressed groups with diet-dependent abundance shifts, add nuance to understanding microbial dynamics under stress.

A limitation of the present study is that the physiological mechanisms potentially linking the observed microbiome changes to host intestinal health were not directly measured. In particular, biomarkers commonly used to assess intestinal barrier integrity, systemic inflammation, oxidative stress, or intestinal histomorphology were not available within the scope of this pilot experiment. The current findings therefore primarily describe microbiome- and network-level responses to heat stress and dietary modulation and future studies integrating microbiome analyses with host physiological markers and intestinal morphology will be necessary to clarify the mechanistic links between microbial community restructuring and host gut health under thermal stress conditions.

## Conclusion

- In summary, across experimental phases, feed efficiency diverged under sustained heat: the heat-stressed control showed a clear deterioration in feed conversion, whereas the thermoneutral control continued to improve; both supplemented diets partially attenuated this penalty despite similar terminal body weight.
- Microbiome readouts only partially mirrored these trends, indicating that not all microbial shifts translate directly into growth parameters. Nevertheless, such alterations do occur, and under prolonged heat exposure, e.g., pigs subjected throughout the ~ 3-month fattening period, they may cumulatively precipitate clinically relevant health complications. Chronic heat was associated with a modest loss of Shannon diversity, a lower Firmicutes: Bacteroidetes ratio, and fewer SCFA producers, features consistent with reduced fermentative capacity, while supplementation, particularly D2, partially restored these attributes. These microbial alterations provide a plausible mechanistic link to the observed decline in feed efficiency, as reduced fermentative capacity may limit energy harvest, whereas the partial restoration under supplementation aligns with the improved feed conversion outcomes.
- Opportunistic taxa tended to expand with prolonged heat exposure, an effect blunted by both diets, more consistently by D2.
- Guild-level indices showed coherent shifts: aerotolerance declined, LPsi remained negative across groups (SCFA-dominant), and BPi increased under D2, indicating higher butyrate capacity.
- Genus-level correlations indicated strong group specificity in microbiota-host interactions, largely driven by HSP70, with only a limited number of taxa exhibiting the strongest positive and negative associations.
- Network analysis further showed reduced modularity and slightly higher connectivity with heat stress; D1 increased modularity with lower density, whereas D2 increased both modularity and network density, consistent with greater ecological robustness. Erysipelotrichaceae emerged as a core keystone across conditions.

## Materials and methods

### Animals and housing

Thirty-six DanBred hybrid barrows were sourced from a commercial pig farm and transported to animal research facility of the University of Debrecen (Hungary). Upon arrival, all animals were individually weighed and ear-tagged for identification. They were then randomly allocated into four treatment groups across 12 pens, ensuring similar average live weights within each group. In the thermoneutral zone, three pens housed three animals each, totaling nine pigs. The high-temperature zone, where elevated temperature was sustained for 24-hours, included nine pens, each housing three pigs, for a total of 27 animals. All pens were bedded with wood shavings as the flooring material. This research program was conducted with the approval of the Animal Ethics Committee of the University of Debrecen under the permit number 9/2019/DEMÁB.

### Experimental periods

The experimental setup consisted of two environmental zones a thermoneutral zone and a high temperature zone.Upon arrival, all animals underwent a 7-day adaptation period (**adaptation phase**) in a thermoneutral environment to acclimate to the new housing conditions and the basal diet. During this time, all treatment groups were fed the same standard diet to ensure uniform nutritional adaptation.

Animals in thermoneutral room were kept in the same condition through the experiment. In the case of high temperature room following initial period, the temperature was gradually increased over one week until reaching the target of 30 °C (**conditioning phase**). This gradual increase served as a thermal adaptation phase for the heat-stressed groups.

The **experimental phase** commenced after the acclimation and conditioning period and lasted for two weeks, during which animals were maintained under their assigned thermal and dietary treatment conditions. Heat stress was applied continuously, with elevated ambient temperature sustained for 24 h per day without diurnal fluctuations.

### Dietary treatments

Three dietary treatments were employed in this study. The feed formulations and their respective chemical compositions have been previously published [89]. Briefly, the basal diet (BD) was based on corn and soybean meal, and its energy and nutrient content was formulated in accordance with the recommendations of the NRC (2012) [90]. for pigs weighing between 75 and 100 kg, with an assumed average daily protein deposition of 155 g.

The basal diet included standard levels of vitamin E, vitamin C, zinc (Zn), and selenium (Se) as specified by the NRC (2012) [90]. In addition to the basal diet, two experimental diets were developed. These were designated as diet 1 (D1) and diet 2 (D2) and contained increasing levels of micronutrient supplementation.

For vitamin C, although not required by NRC (2012) [90] guidelines for growing-finishing pigs, supplementation was included based on DSM’s Optimum Vitamin Nutrition (OVN^®^), recommendations [91]. Diet 1 contained 150 mg/kg, and diet 2 contained 300 mg/kg of vitamin C. For vitamin E, the OVN^®^ range of 64–105 mg/kg guided the decision to increase the basal level by 30 mg/kg in each step, resulting in 41 mg/kg in D1 and 71 mg/kg in D2.

Regarding zinc, the maximum permissible level in the EU is 150 mg/kg. Accordingly, zinc levels were set at 50 mg/kg in the basal diet, 100 mg/kg in D1, and 150 mg/kg in D2. For selenium, considering toxicity concerns and EU regulations, selenium supplementation was carefully adjusted. The basal diet contained 0.16 mg/kg Se, while D1 and D2 were enriched to 0.21 mg/kg and 0.26 mg/kg, respectively.

Animals housed in the thermoneutral environment received only the basal diet. In the high-temperature room (“heat stress room”), pigs were divided into three dietary treatment groups: basal diet, diet 1, and diet 2. Throughout the experiment, all animals had *ad libitum* access to feed and water.

### Animal house measurements

During the experimental phase, environmental conditions in the thermoneutral room were maintained at a constant temperature of 19.5 ± 1.8 °C, and a relative humidity of 86.5 ± 8.4%. This resulted in a Temperature-Humidity Index (THI) [92] of 66.4 ± 1.6. Room conditions in a high-temperature environment were kept constant during the experimental phase at 28.9 ± 0.9 °C with 60.4 ± 4.3% humidity over 24 h (THI index 78.4 ± 1.7). Based on these results, the fattening pigs in our experiment were housed under normal and emergency conditions, in terms of heat-stress [93]. Ambient temperature and relative humidity were continuously measured in each room using a calibrated temperature and humidity data logger (Testo 174 H; Testo SE & Co. KGaA, Lenzkirch, Germany). The data were recorded every day at 7:30 am and 3:00 pm. Upon arrival, animals weighed 65.1 ± 2.81 kg. Body weight and feed conversion ratio were recorded across experimental phases. The apparent total tract digestibility (ATTD) was determined as described in the previous publication Ortega et al. [19].

### Feces sample collection

Feces samples were collected from all animals individually, using rectal stimulation by gently inserting a sterile cotton swab 20–30 mm into the rectum and making small circular, back-and-forth movements for up to 2 min to induce defecation [94, 95]. If defecation occurred, 10 g fecal material was collected in sterile tubes. By the end of the experiment, a total of 93 fecal samples were collected, with missing samples attributable to unsuccessful collection attempts. All samples were kept in liquid nitrogen until transported to the laboratory of the University of Debrecen, Faculty of Agricultural and Food Sciences and Environmental Management, Center for Complex Systems and Microbiome Innovations. The samples were stored at -80 °C until processing.

### Sample preparation and DNA extraction

Bacterial cell suspensions were prepared from pigs’ feces samples following the protocol as previously described by our group [96, 97].

DNA extraction was performed using the MagNa Pure 24 System (Roche Applied Sciences, Germany), following the Pathogen 200 protocol. Briefly, cell lysis was initiated by adding 150 µL of MagNA Pure Bacteria Lysis Buffer (BLB) buffer (Roche Applied Sciences, Germany) and 30 µL of Proteinase K solution (Lot No. 03115879001, Roche Applied Sciences, Germany) to 150 µL of bacterial cell suspension, followed by thorough vortexing (FV-2400 Minicentrifuge-Vortex Microspin, Biosan, Latvia, Riga). The samples were then incubated at 65 °C for 10 min at 300 rpm in an Eppendorf ThermoMixer^®^ C (Eppendorf, Hamburg, Germany). This was followed by a second incubation step at 95 °C for 10 min, after which the samples were immediately cooled on ice.

Following cooling, the samples were centrifuged at 16,000 × g for 1 min at 4 °C, and the supernatant was transferred to a new tube. A volume of 200 µL was then loaded into the MagNa Pure 24 System cartridge, and the Pathogen 200 protocol was executed.

DNA concentrations were determined fluorometrically using the Qubit^®^ dsDNA High Sensitivity Assay Kit (Invitrogen by Thermo Fisher Scientific, Cat. 2600187) in conjunction with the Qubit^®^ 4.0 Fluorometer (Thermo Fisher Scientific, USA), and were also measured spectrophotometrically using a NanoDrop™ Spectrophotometer (Thermo Fisher Scientific, USA) to assess purity and concentration.

### Library preparation and 16 S sequencing

Standard library preparation was carried out using Illumina’s 16 S Metagenomic Sequencing Library Preparation protocol (15044223 Rev. B, San Diego, CA, USA). The V3–V4 hypervariable regions of the bacterial 16 S rRNA gene were amplified, generating ~ 460 bp amplicons with the universal primers 341 F (5′-CCTACGGGNGGCWGCAG-3′) and 785R (5′-GACTACHVGGGTATCTAATCC-3′), both flanked by Illumina overhang adapter sequences (forward: 5′-TCGTCGGCAGCGTCAGATGTGTATAAGAGACAG-3′; reverse: 5′-GTCTCGTGGGCTCGGAGATGTGTATAAGAGACAG-3′) (Sigma-Aldrich, Missouri, USA).

PCR amplification was performed using 2× KAPA HiFi HotStart ReadyMix, followed by dual indexing of the amplicons using the Nextera XT Index Kit (FC-131-1001/2, Illumina). Amplicon purification and size selection (~ 580–630 bp) were conducted with KAPA Pure Beads, according to the manufacturer’s protocol (KR1245-v3.16). PCR product verification was done using Agilent D1000 ScreenTape (5067–5582) and D1000 reagents (5067–5583).

Libraries were quantified by qPCR, normalized based on amplicon size, and pooled in equimolar concentrations. A final volume of 5 µl from a 4 nM pooled library was used for sequencing. The library pool was denatured with 0.2 M NaOH, diluted to 8 pM, and sequenced using an Illumina MiSeq platform with the MiSeq Reagent Kit v3 (618-cycle, MS-102-3003), following standard protocols. Paired-end sequencing (2 × 301 bp) was conducted, including a 5% PhiX spike-in (PhiX Control Kit v3, FC-110-3001) as an internal quality control.

### Description of sequencing results

The sequencing resulted in 124,850 ± 22,853 reads per sample on average. After denoising, merging paired-end reads, and removing chimeras, 2,660 ± 945 reads remained, resulting in a total of 2,724 ASVs, 96 ± 29 per sample on average.

### Metagenomic data processing

Demultiplexing of the paired-end reads and FASTQ file generation were carried out using Illumina’s BaseSpace software. Downstream processing and analysis of the sequencing data were performed with QIIME 2 (version 2024.10) [98]. Adapter sequences (CTGTCTCTTATACACATCT) were identified and removed from the 3′ ends of the reads using the Cutadapt plugin within the QIIME 2 environment. Quality control, including trimming, filtering, and chimera removal, was performed using DADA2. Amplicon sequencing variants (ASVs) were inferred.

For trimming parameters, both the forward and reverse reads were cropped by 1 base at the start and truncated at 300 bases. Multiple-sequence alignment was conducted using MAFFT. Taxonomic classification of the resulting ASVs was performed using a naïve Bayes classifier trained on the SILVA reference database (version 138_2). The classifier was tailored to the primer pair used for amplification (341 F and 785R), targeting the V3-V4 region of the bacterial 16 S rRNA gene. Predicted functional profiles were generated from ASVs using PICRUSt2, which includes phylogenetic placement of ASVs, hidden-state prediction of gene families, and pathway-level inference [99]. All results are reported as predicted functional features rather than directly measured functions. Predicted gene families and pathways were annotated by mapping to Kyoto Encyclopedia of Genes and Genomes (KEGG) identifiers for functional classification. No KEGG pathway maps or other copyrighted KEGG content were reproduced [100].

### Quantitative PCR analysis of cytokines and heat shock proteins

Quantitative PCR was used to quantify the expression of cytokine and heat shock protein genes in pig white blood cells and jejunum tissue following reverse transcription of RNA into cDNA as reported in our companion study [19]. Gene-specific, intron-spanning primers were applied, and expression levels were measured in triplicate using a real-time PCR system, with normalization to validated housekeeping genes. Relative gene expression was calculated using the ΔCt method.

### Statistical analysis and data visualization

The Wilcoxon rank-sum test was applied to statistically compare continuous variables. All statistical tests were two-tailed, with a significance level set at *p* < 0.05. Continuous variables were reported as the mean ± standard deviation. Bar plots, box plots, polar plots, bubble plots, line plots, pie charts, and area plots were generated using the ‘ggplot2’ R package (version 3.5.1) [101]. The Shannon index was calculated to assess species richness and evenness, based on the species profile, using QIIME2 software [98, 102]. PCoA was conducted to analyze the differences in microbial community by using weighted UniFrac distances [103]. Bray-Curtis distance was calculated using vegan R package [104]. To identify taxa most strongly associated with community-level ordination structure, species vectors were fitted onto the canonical correspondence analysis (CCA) ordination using the envfit function (vegan R package, /version 2.5-4/ with 999 permutations) [104]. The resulting r² values indicate the proportion of variance in the constrained ordination space explained by each taxon vector, and significance was assessed by permutation test. Only taxa with a permutation-derived *p*-value < 0.05 were retained for interpretation. A differential heat tree was created with the Metacoder R package [105]. The volcano plot was generated with ggplot2 (version 3.5.1) and ggrepel (version 0.9.6) [101, 106], and the heatmap was generated with pheatmap R package (version 1.0.12) [107]. Venn diagrams were assessed with limma R package (version 3.60.3) [108]. For microbial network analysis, the NetCoMi R package (v.1.2.0) was used with centered log-ratio (clr) normalization, and Pearson correlation estimates [109]. Pearson correlation coefficients were calculated to assess linear relationships between variables using R.

## Supplementary Information

Below is the link to the electronic supplementary material.

Supplementary Material 1

Supplementary Material 2

Supplementary Material 3

Supplementary Material 4

Supplementary Material 5

## Data Availability

All sequence data used in the analyses were deposited in the Sequence Read Archive (SRA) (http://www.ncbi.nlm.nih.gov/sra) under PRJNA1253913.
