# Supplementary figures and images for: Alleviation of heat stress-induced microbial dysbiosis in pigs through dietary supplementation with vitamins and trace elements
Source: Anim Microbiome. 2026 Apr 21;8:75. doi: 10.1186/s42523-026-00575-4 (PMC13245032; doi:10.1186/s42523-026-00575-4)

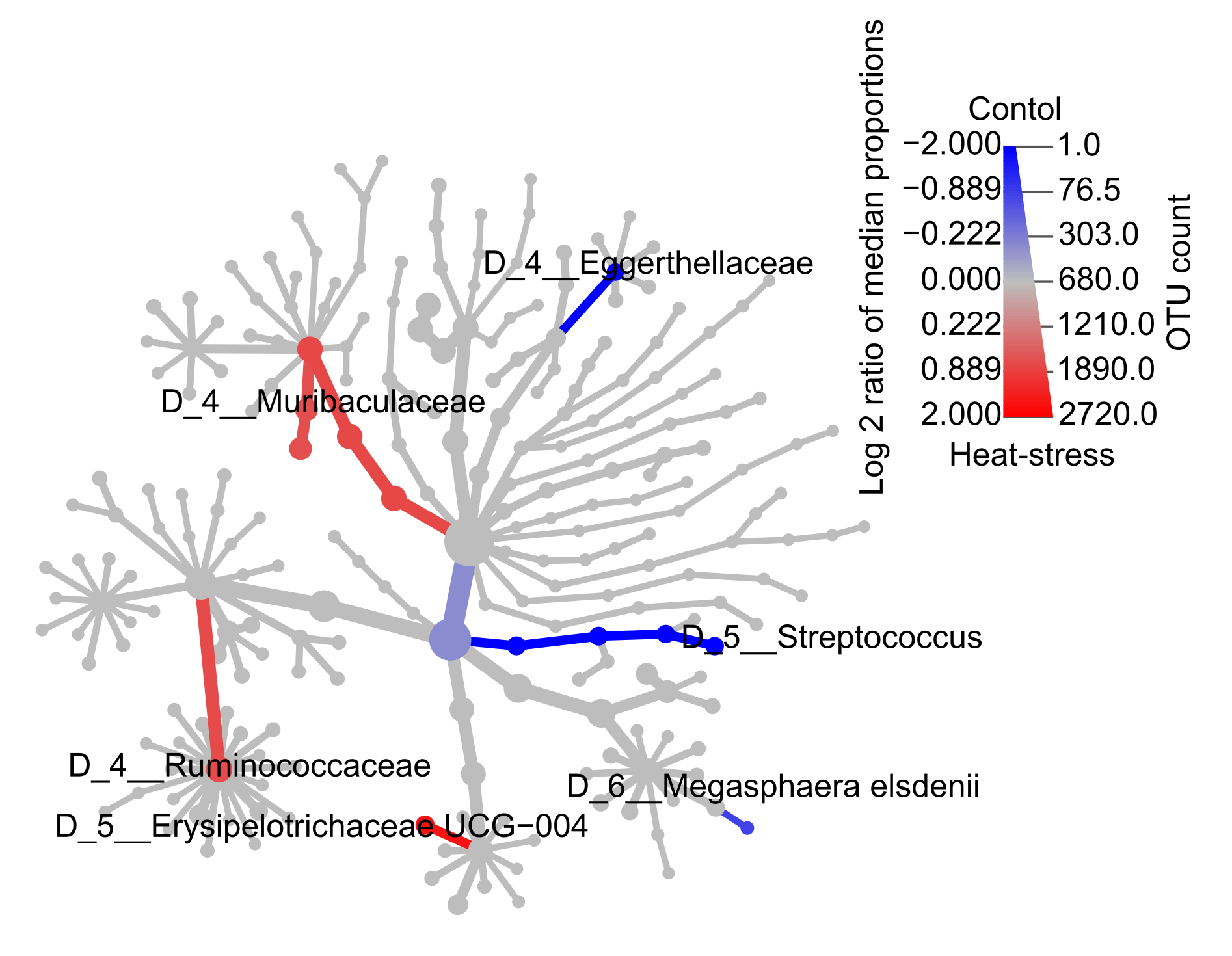

Supplement: Supplementary file 2 — Supplementary Material 2 [file 42523_2026_575_MOESM2_ESM.png]

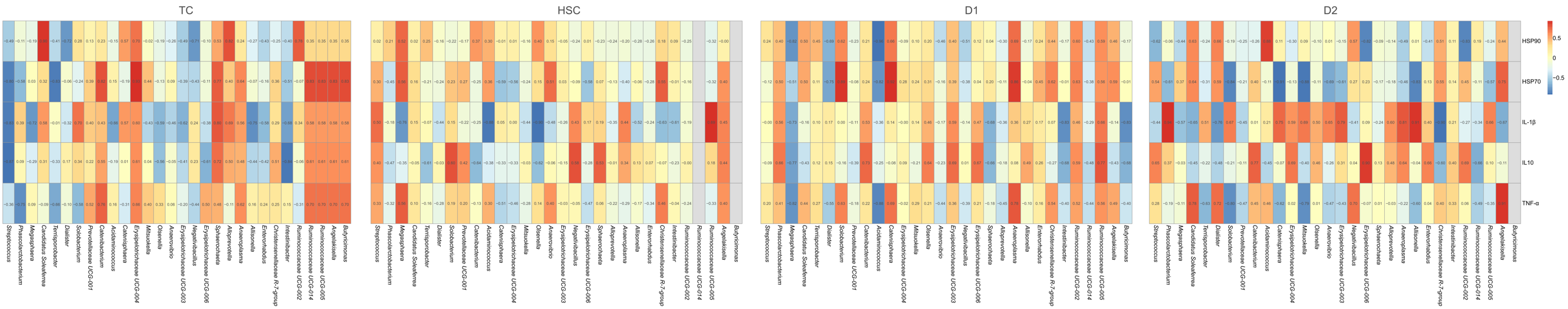

Supplement: Supplementary file 3 — Supplementary Material 3 [file 42523_2026_575_MOESM3_ESM.png]
